# Functional Characterization of CYP94-Genes and Identification of a Novel Jasmonate Catabolite in Flowers

**DOI:** 10.1371/journal.pone.0159875

**Published:** 2016-07-26

**Authors:** Viktoria Bruckhoff, Sven Haroth, Kirstin Feussner, Stefanie König, Florian Brodhun, Ivo Feussner

**Affiliations:** 1 Georg-August-University Goettingen, Albrecht-von-Haller Institute for Plant Sciences, Department of Plant Biochemistry, Goettingen, Germany; 2 Georg-August-University Goettingen, Goettingen Center for Molecular Biosciences (GZMB), Department of Plant Biochemistry, Goettingen, Germany; Oak Ridge National Laboratory, UNITED STATES

## Abstract

Over the past decades much research focused on the biosynthesis of the plant hormone jasmonyl-isoleucine (JA-Ile). While many details about its biosynthetic pathway as well about its physiological function are established nowadays, knowledge about its catabolic fate is still scarce. Only recently, the hormonal inactivation mechanisms became a stronger research focus. Two major pathways have been proposed to inactivate JA-Ile: i) The cleavage of the jasmonyl-residue from the isoleucine moiety, a reaction that is catalyzed by specific amido-hydrolases, or ii), the sequential oxidation of the ω-end of the pentenyl side-chain. This reaction is catalyzed by specific members of the cytochrome P450 (CYP) subfamily CYP94: CYP94B1, CYP94B3 and CYP94C1. In the present study, we further investigated the oxidative fate of JA-Ile by expanding the analysis on *Arabidopsis thaliana* mutants, lacking only one (*cyp94b1*, *cyp94b2*, *cyp94b3*, *cyp94c1*), two (*cyp94b1xcyp94b2*, *cyp94b1xcyp94b3*, *cyp94b2xcyp94b3*), three (*cyp94b1xcyp94b2xcyp94b3*) or even four (*cyp94b1xcyp94b2xcyp94b3xcyp94c1*) CYP94 functionalities. The results obtained in the present study show that CYP94B1, CYP94B2, CYP94B3 and CYP94C1 are responsible for catalyzing the sequential ω-oxidation of JA-Ile in a semi-redundant manner. While CYP94B-enzymes preferentially hydroxylate JA-Ile to 12-hydroxy-JA-Ile, CYP94C1 catalyzes primarily the subsequent oxidation, yielding 12-carboxy-JA-Ile. In addition, data obtained from investigating the triple and quadruple mutants let us hypothesize that a direct oxidation of unconjugated JA to 12-hydroxy-JA is possible *in planta*. Using a non-targeted metabolite fingerprinting analysis, we identified unconjugated 12-carboxy-JA as novel jasmonate derivative in floral tissues. Using the same approach, we could show that deletion of *CYP94*-genes might not only affect JA-homeostasis but also other signaling pathways. Deletion of *CYP94B1*, for example, led to accumulation of metabolites that may be characteristic for plant stress responses like systemic acquired resistance. Evaluation of the *in vivo* function of the different CYP94-enzymes on the JA-sensitivity demonstrated that particularly CYP94B-enzymes might play an essential role for JA-response, whereas CYP94C1 might only be of minor importance.

## Introduction

In plants a diverse set of different plant-specific hormones, the phytohormones, orchestrates different metabolic settings of growth, development and defense. In response to wounding and necrotrophic pathogen attack, the plant hormone jasmonyl-isoleucine (JA-Ile) plays a key-role as it activates a rapid metabolic defense answer to those stress stimuli [1].

Its biosynthetic pathway may start in the plastid where a lipase releases 18:3(n-3) (x:y(n-z) denoting a fatty acid with x carbons and y double bonds in position z counting from the methyl end) from the inner plastidial envelope membrane. Here, a 13*S*-lipoxygenase oxidizes the free 18:3(n-3) to 13*S*-hydroperoxy octadecatrienoic acid. In the following reaction steps this product is converted to *cis*(+)-12-oxo phytodienoic acid (*cis*(+)OPDA) by the successive action of allene-oxide synthase and allene-oxide cyclase [2]. In the peroxisome *cis*(+)OPDA is reduced, activated and processed by three rounds of β-oxidation finally yielding (+)-7-*iso*-JA (for reasons of simplicity this compound will be abbreviated as JA in the following sections).

Up to now six different metabolic routes are known by which JA may be further processed, yielding a highly diverse set of JA-derived metabolites [1, 3]. An important modification of JA is its conjugation to amino acids, a reaction that is catalyzed by the jasmonate amido synthetase JASMONATE RESISTENT1 (JAR1) [4]. However, only for the JA-Ile conjugate a gene-regulating activity has been observed: In the presence of JA-Ile the CORONATINE INSENSITIVE 1 (COI1) receptor interacts with JASMONATE ZIM-DOMAIN (JAZ) proteins. Those proteins block the binding of different transcription factors (as for example MYC2) and thus serve as transcriptional repressors of JA-responsive genes. This binding event of COI1-JA-Ile and JAZ proteins leads to the proteolytic degradation of the JAZ-repressors and thereby ultimately allows the transcription of those genes [5].

**Fig 1:**
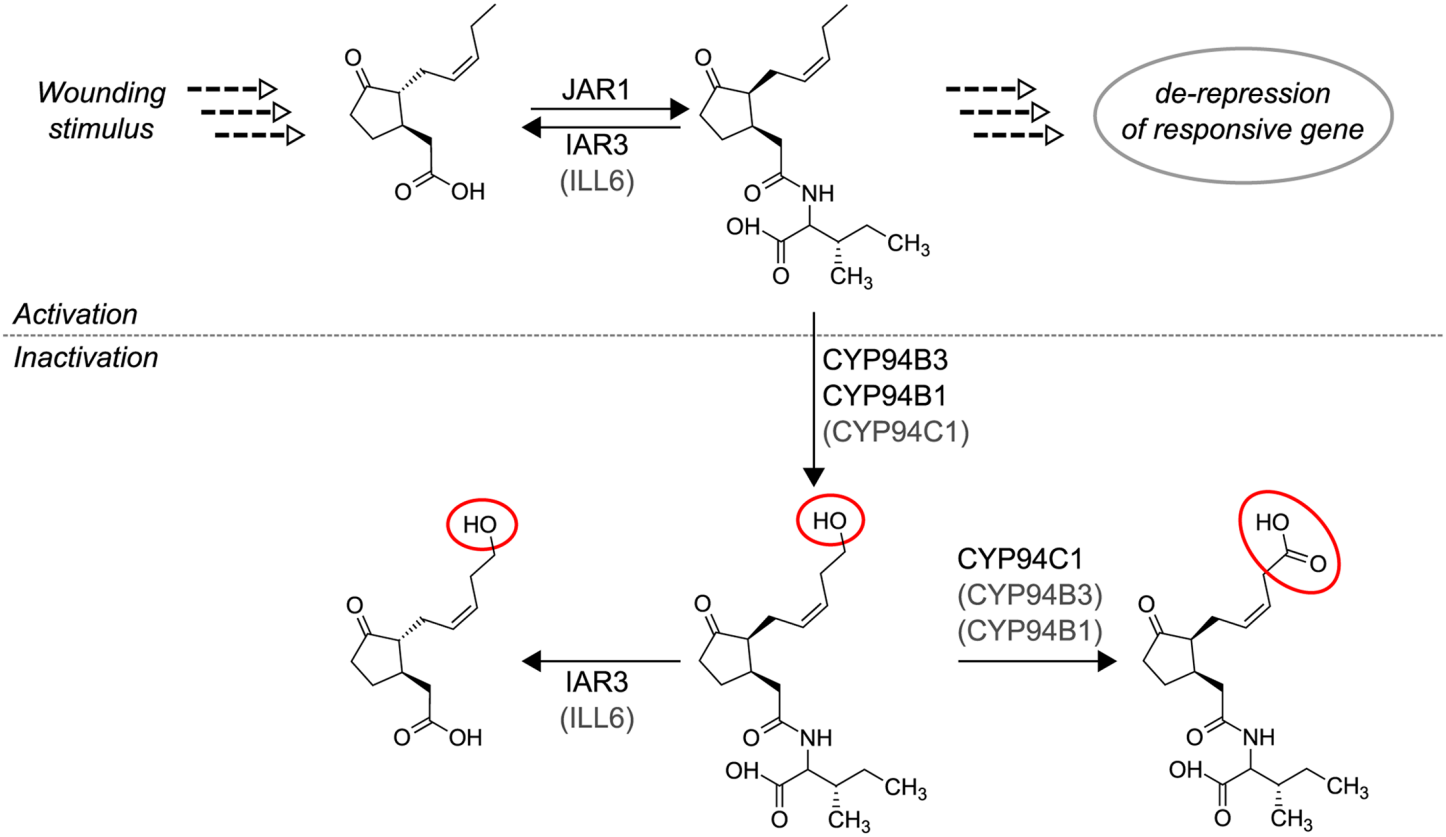
Pathway of JA activation and inactivation. Plant wounding induces the conversion of 18:3(n-3) to JA (for details see text). JA might be conjugated to Ile yielding JA-Ile, a reaction catalyzed by JAR1. JA-Ile is the bioactive phytohormone, which can be perceived by the SCF^COI^-complex leading to the de-repression/induction of JA-responsive genes. Inactivation of JA-Ile signaling can be achieved via two possible routes: either by the enzymatic activity of the amido-hydrolases ILL6 and IAR3 that catalyze the hydrolytic cleavage of JA-Ile, or by enzymatic activity of distinct members of the cytochrome P450 subfamily CYP94 (*i*.*e*. CYP94B1, CYP94B3 and CYP94C1) that catalyze the sequential ω-oxidation of JA-Ile to 12-hydroxy-JA-Ile and 12-carboxy-JA-Ile. Although all three mentioned CYP94-enzymes have the capacity to catalyze the hydroxylation (mono-oxygenation) as well as the carboxylation (double oxygenation), they exhibit distinct catalytic specificities. Beside JA-Ile, oxidized JA-Ile derivatives may also serve as substrate for IAR3 (and Ill6) *in planta*. JAR, JASMONATE RESISTENT1; IAR3, IAA-ALA-RESISTENT3; ILL6, IAA-LEU RESISTENT-like6.

While the biosynthetic pathway leading to the formation of JA-Ile has been investigated intensively over the last decades, only recent studies focused on its inactivation and catabolic fate. It was demonstrated, that inactivation can be achieved by two possible ways: either, by hydrolytic cleavage of the jasmonyl-residue from the Ile-moiety (a reaction which is often referred to as “deconjugation”) or by a sequential ω-oxidation of the pentenyl site-chain of JA-Ile (a process that is often referred to as “oxidative inactivation”). The enzymes responsible for the catalytic deconjugation of JA-Ile have recently been identified by several groups as members of the indole-3-acetic acid (IAA) amido-hydrolase enzyme family [6–9]. It was shown that in particular IAA-ALA-RESISTENT3 (IAR3) and IAA-LEU RESISTENT-like6 (ILL6) are essential key-players for sustaining JA-homeostasis *in planta* [6, 7]. The oxidative inactivation of JA-Ile, on the other hand, was found to be catalyzed by three members of the cytochrome P450 (CYPs) subfamily CYP94 [10–14]. Although all three enzymes are capable to sequentially oxidize JA-Ile to 12-hydroxy-JA-Ile and further to 12-carboxy-JA-Ile, it was shown that CYP94B1 and CYP94B3 preferentially catalyze the hydroxylation (mono-oxygenation) reaction [10, 11, 13, 15]. On the contrary, CYP94C1 is more specific for catalyzing the following sequential double oxygenation reaction that yields first 12-oxo-JA-Ile and finally 12-carboxy-JA-Ile [13] (*cf*. Fig 1).

In order to expand our knowledge on the oxidative fates of JA-Ile, we compared each of the four single mutants (*cyp94b1*, *cyp94b2*, *cyp94b3*, *cyp94c1*) with the *CYP94B* related double (*cyp94b1xcyp94b2*, *cyp94b1xcyp94b3*, *cyp94b2xcyp94b3*), triple (*cyp94b1xcyp94b2xcyp94b3*) and quadruple (*cyp94b1xcyp94b2xcyp94b3xcyp94c1)* loss-of-function mutants. We were particularly interested on the one hand in the metabolic changes occurring at the early time-point after wounding and focused on the time-dependent alterations of JA, JA-Ile and their ω-oxidized derivatives. A recent study by Widemann and co-workers suggested that CYP94-enzymes play an important role during flower opening [14]. We followed up on this idea and analyzed the influence of *CYP94* deletions on flower development on a physiological as well as on a metabolic level. For this purpose, we used a non-targeted metabolite fingerprinting approach that allowed the identification of metabolites that have not been observed before in the context of JA homeostasis. Our results thus demonstrate that *CYP94*-genes are not only essential for sustaining JA homeostasis but also affect other metabolic pathways.

## Material and Methods

### Plant material and growth conditions

For all experiments, we used *Arabidopsis thaliana* ecotype Col-0 as wild-type reference. T-DNA insertion lines [16] *cyp94b1* (SAIL_502_G01), *cyp94b2* (SM_3_37400), *cyp94b3* (SALK_018989) [10] and *cyp94c1* (SALK_011290) [17] were obtained from the Nottingham Arabidopsis Stock Centre and genotyped using primers presented in S1 Table [16]. *cyp94b3* (SALK_018989) and *cyp94c1* (SALK_011290) have been described recently [10] [17]. We confirmed the knock-out of all single T-DNA-insertion mutants by semi-quantitative RT-PCR (S1 Fig). Double, triple and quadruple mutants were generated by crossing the respective homozygous plant lines. PCR-genotyping of the resulting F2 generation was performed to confirm successful crossing. All plants were grown as described in [18]. Briefly, Arabidopsis seeds were sown on steamed soil (80°C, 8 h) and stratified for 2 d at 4°C in the dark in order to synchronize germination. Plants were cultivated at 22°C and 60% humidity in a Percival growth chamber (Percival Scientific, USA) under long day conditions (photoperiod: 16 h light/8 h dark) with a light intensity of 120–150 μmol m^-2^s^-1^.

### Statistical analysis

Data were statistically analyzed by Student’s Test and by the one-way analysis of variance (ANOVA) using the Tukey-post-hoc test (*p*<0.05). We employed for these methods the Origin Pro 8.5 and R-Studio software (Agricolae package).

### Histochemical promoter-β-Glucoronidase staining

As the exact positions of the *CYP94*-genes have not been reported previously we used a region of 2 kbp upstream of the start codon as a start. This length is suggested to cover most promoters and some regulatory regions upstream from the start codon [16]. Furthermore, it is known that sometimes regulatory regions also exist downstream from the start of the coding region [19]. Consequently, we generated for some *CYP94-*genes two constructs, one consisting of the respective promoter region and one that contained additional 30–40 codons downstream from the start position. The primers used for this approach are listed in Table 1. The molecular cloning procedure and promoter-β-glucoronidase-assay were performed as described in [18]. Staining was performed with 2 mM x-Gluc (Glycosynth, UK).

**Table 1:** List of Primers used for cloning of promoter-β-glucoronidase fusion constructs.

Primer name	Nucleotide sequence (5‘->3‘)
PromCyp94B1for	ATTCTCCAACCAATACTTTGTCGTCC
PromCyp94B1rev1	TTTGGATTCTTGGGTTTCTTGTTTGG
PromCyp94B1rev2	TTTTGTTGAAGGAGAGAATAGAGCCG
PromCyp94B2for	TTTCTCAATATTTTATTTTAGCCTC
PromCyp94B2rev1	TGTTGATGAGCTTCAAAACGAC
PromCyp94B2rev2	ATGAAATTAAGCATCCGATAACC
PromCyp94B3for	ATGAAGACAAAAAACGGTTGTTCC
PromCyp94B2rev1	ATTGTTTAATTGTTTTTTGTTCTTGGT
PromCyp94B3rev2	TTTTGTTGAAAGAGAGTATGGAGC
PromCyp94C1for	ATGGAAATTTTAATTCGAGTTATAGC
PromCyp94C1rev1	TGTTGATGAGGAGACAAAGAGAAAG
PromCyp94C1rev2	GATTGATGAAGTCTTTTTTCCAGC

### Semi-quantitative reverse transcription

RNA was isolated from various *A*. *thaliana* tissues, including leaves, roots, stems, flowers, petioles and siliques, using the Plant-RNA Kit from Sigma-Aldrich (Germany) according to the manufacturer’s advice. 1 μg of isolated RNA was treated with DNaseI (30 min, 37°C). For reverse transcription of RNA into cDNA we used the RevertAid H Minus Reverse Transcriptase and oligo(dT)18 primer according to manufacturer’s instructions (MBI Fermentas, Germany). 1 μL of the resulting cDNA was used for the polymerase-chain reaction employing the RedTaq polymerase (Sigma-Aldrich, Germany). Primer used for this analysis are listed in Table 2.

**Table 2:** List of Primers used for semi-quantitative reverse transcriptase polymerase chain reaction.

Primer name	Nucleotide sequence (5‘->3‘)
Cyp94B1_LP	TCGGTTCTTCCTCAAACCAC
Cyp94B1_RP	GTTTACGCCGTGTGGAAAGT
Cyp94B2_LP	CCATCTCTTCCCCAAGACAA
Cyp94B2_RP	CCATAACTGCGTCGATGAGA
Cyp94B3_LP	GGCACAATCTCAAACCGACT
Cyp94B3_RP	TTTTATCATGGCGGGAAGAG
Cyp94C1_LP	CCGACAATCCTGGTTCTGTT
Cyp94C1_RP	GCCATCTCTTTCCCGATACA
AtActin_for	GCTGGATTCGCTGGAGATGA
AtActin_rev	AGGTCTCCATCTCTTGCTCG

### Root growth inhibition assays

Seeds of *A*. *thaliana* were surface sterilized and sown on solidified ½ MS medium [20] containing either 0 or 10 μM JA in square Petri-dishes (10 x 10 cm). 10 to 15 seeds per plant line were seeded individually per plate. Plates were kept upright under long day or continuous light conditions depending on the experimental setup. The root length was determined after 7 d, after 10 d and after 14 d depending on the experimental setup.

### Plant wounding and metabolite extraction

Plants were wounded according to the procedure described in [21] and metabolites were extracted as described previously by [22] at different time-points after wounding. Tissue samples were frozen in liquid nitrogen and homogenized. We used 200 mg of ground roots or leaves, or 50 mg of homogenized flowers for each analysis. Frozen plant material was mixed with 750 μL ice-cold methanol and 2.5 mL methyl-*tert*-butyl ether (MTBE). For quantification we added 10 ng D6-JA, 30 ng D5-*cis*(+)OPDA, 10 ng D3-JA-Leu (kindly provided by Dr. Otto Miersch, IPB, Halle/Saale, Germany), 20 ng D5-indole acetic acid (IAA) (Eurisotop, Germany), 20 ng D5-Zeatin, 10 ng D3-GA3 (OlChemIm Ltd, Czech Republic), 100 ng 2-oxothiazolidine-4- carboxylic acid (OxoRA) as internal standards. After 60 min rapid shaking at 4°C in the dark, 600 μL water were added and samples were further incubated for 10 min. The mixture was centrifuged for 15 min at 1200 x g at 4°C and the resulting two phases were divided: The upper MTBE phase containing mainly hydrophobic metabolites was evaporated under a stream of nitrogen to dryness and re-dissolved in a solution consisting of acetonitirile/water/acetic acid (20/80/0.1, *v/v/v*); for non-targeted metabolite fingerprinting analysis, this extract was re-dissolved in chloroform/methanol/water (60/30/4, *v/v/v*), while the extract from the lower methanol-water phase was re-dissolved in methanol/acetonitrile/water (10/10/120, *v/v/v*).

### Determination of JA-, JA-Ile and their oxidized derivatives using LC/MS

Different jasmonate-derivatives were analyzed according to a procedure described in [23] employing an Agilent 1100 HPLC system (Agilent Technologies, USA) equipped with an EC 50/2 Nucleodure C18 gravity 1.8 μm column. The system was coupled to an Applied Biosystems 3200 hybrid triple quadruple/linear ion trap mass spectrometer. Nanoelectrospray ionization (NanoESI) was performed using a chip ion source (Advion Biosciences, USA). For metabolite separation a binary gradient system was used consisting of solvent A, water/acetic acid (100/0.1, *v/v*) and solvent B, acetonitrile/acetic acid (100/0.1, *v/v*). The gradient was as follows: 5% solvent B for 1 min, followed by a linear increase of solvent B up to 95% within 10 min and an isocratic run at 95% solvent B for 4 min. To re-establish starting conditions a linear decrease to 5% B within 2 min was performed, followed by 10 min isocratic equilibration at 5% B. The flow rate was 0.3 ml min-1. For stable nanoESI, 130 μl min^-1^ of 2-propanol/acetonitrile/water/acetic acid (70/20/10/0.1, *v/v/v/v*) delivered by a 2150 HPLC pump (LKB, Sweden) were added directly after the column via a mixing tee valve. Through another post column splitter 790 nl min^-1^ of the eluent were directed to the nanoESI chip. The ionization voltage was set to -1.7 kV. Analytes were ionized negatively and measured in a scheduled measurement. The measuring mode was a single-reaction monitoring mode to ensure high specificity and sensitivity for the different jasmonate-derivatives. Mass transition 137/93 for salicylic acid (declustering potential (DP) -25 V, entrance potential (EP) -6 V, cell entrance potential (CEP) -10 V, collision energy (CE) -20 V, cell exit potential (CXP) -10 V); 209/59 JA (DP -30 V, EP -4.5 V, CEP -14 V, CE -24 V, CXP -6 V); 215/59 D6-JA (DP -35 V, EP -8.5 V, CEP -14 V, CE -24 V, CXP -6 V); 225/59 11/12-OH-JA (DP -35 V, EP -9 V, CEP -14 V, CE -28 V, CXP -6 V); 225/165 11/12-OH-JA-1 (DP -35 V, EP -9 V, CEP -14 V, CE -16 V, CXP -2 V); 322/130 JA-Ile/Leu (DP -45 V, EP -5 V, CEP -14 V, CE -28 V, CXP -2 V); 325/133 D3-JALeu (DP -65 V, EP -4 V, CEP -16 V, CE -30 V, CXP -2 V); 338/130 12OH-JA-Ile (DP -45 V, EP -10 V, CEP -14 V, CE -30 V, CXP -6 V); 352/130 12COOH-JA-Ile (DP -45 V, EP -10 V, CEP -14 V, CE -30 V, CXP -6 V).

The mass analyzers were adjusted to a resolution of 0.7 atomic mass units (amu) full width at half-height. The ion source temperature was at 40°C, and the curtain gas was set to 10 (given in arbitrary units). Quantification of JA, 12-hydroxy-JA and JA-Ile was carried out using a calibration curve of intensity (*m/z*) ratios. The ratios were calculated of [unlabeled]/[deuterium labeled] vs. molar amounts of unlabeled (0.3–1000 pmol). Relative quantification of 12-hydroxy-JA-Ile and 12-carboxy-JA-Ile was performed using deuterated JA-Ile as internal standard.

### Non-targeted metabolic fingerprinting

Metabolic fingerprinting was performed as described elsewhere with some modifications [24]. Samples of two pools of flowers of two independent experiments each were analyzed. The analysis was performed twice for each sample by Ultra Performance Liquid Chromatography (UPLC) coupled with a photo diode array (PDA) detector and an orthogonal time-of-flight mass spectrometer (TOF-MS). For LC an ACQUITY UPLC BEH RP18 column (1 x 100 mm, 1.7 μm particle size) was used for the samples of the non-polar extraction phase and an ACQUITY UPLC HSS T3 (1 x 100 mm, 1.8 μm particle size) for these of the polar extraction phase. LC was performed at a temperature of 40°C, a flow rate of 0.2 ml min^-1^ and with a binary gradient of solvent A (water/formic acid (100/0.1, *v/v*) and solvent B (acetonitrile/formic acid (100:0.1, *v/v*). The following gradient was applied for the analysis of the samples of the polar extraction phase: 0–0.5 min 10% solvent B, 0.5–3 min from 10% to 28% solvent B, 3–8 min from 28% up to 95% solvent B, 8–10 min 95% solvent B and 10–14 min 10% solvent B and for the analysis of the samples of the non-polar extraction phase: 0–0.5 min 46% solvent B, 0.5–5.5 min 46 to 99% solvent B, 5.5–10 min 100% solvent B and 10–13 min 46% solvent B. The TOF-MS was operated in W optics to ensure a mass resolution larger than 10,000 in negative as well as positive electrospray ionization (ESI) mode. Data were acquired by MassLynx 4.1 software in centroided format over a mass range of *m/z* 50–1200 (negative ESI mode) and *m/z* 85–1200 (positive ESI mode) with a scan duration of 0.5 sec and an inter-scan delay of 0.1 sec. The capillary and the cone voltage were maintained at 2,700 V and 30 V and the desolvation and source temperature at 350°C and 80°C, respectively. Nitrogen was used as cone (30 l h^-1^) and desolvation gas (800 l h^-1^). The Dynamic Range Enhancement (DRE) mode was used for data recording. All analyses were monitored by using Leucine-enkephaline ([M-H]^-^ 554.2615 or [M+H]^+^ 556.2771) as well as its ^13^C isotopologue ([M-H]^-^ 555.2615 or its double ^13^C isotopologue [M+H]^+^ 558.2836) as lock spray reference compound at a concentration of 0.5 μg ml^-1^ in acetonitrile/water (50:50, *v/v*) and a flow rate of 30 μl min^-1^. Processing of raw data to data matrices was performed as follows: The raw mass spectrometry data were taken together from one experiment including all samples. With MarkerLynx Application Manager for MassLynx 4.1 software the raw data were processed (peak picking and peak alignment), resulting in four data matrices. The data matrices are according to the polar extraction phase positively or negatively ionized, and for the non-polar extraction phase positively or negatively ionized. Further data processing, comprising ranking and filtering of the data, matching high quality features of the two independent experiments to one representative data set, adduct identification and correction as well as combining of the data matrixes for clustering and visualization, was carried out with the toolbox MarVis (MarkerVisualization, http://marvis.gobics.de, [25, 26]).

The toolbox MarVis includes the subroutines MarVis Filter [27] and MarVis Cluster [28]. First an ANOVA test combined with a multiple testing (Benjamini-Hochberg) was performed to filter and extract features with a false discovery rate (FDR) <10^−4^. Subsequently data of two independent experiments were matched and the selected high quality features were chosen and their masses were adduct corrected according to the following rules: [M+H]^+^, [M+Na]^+^, [M+NH_4_]^+^ for the positive and [M-H]^-^, [M+CH_2_O_2_-H]^-^, [M+CH_2_O_2_+Na-2H]^-^ for the negative ionization mode. Subsequently data sets could be combined, used for visualization by cluster analysis and automated database search [25]. For data base search the following databases were used: KEGG (http://www.genome.jp/kegg), LipidMaps (http://www.lipidmaps.org), Aracyc (https://www.arabidopsis.org/biocyc), Knapsack (http://kanaya.naist.jp/KNApSAcK) and In-house databases. The identity of marker metabolites was confirmed by co-elution with authentic standards and/or by UHPLC-ESI-MS/MS analysis.

### Structure determination of marker metabolites by UHPLC ESI-QTOF-MS

The identity of marker metabolites described by metabolite fingerprinting was confirmed by MS/MS analysis. For that samples were analyzed by LC 1290 Infinity (Agilent Technologies, USA) coupled with an 6540 UHD Accurate-Mass Q-TOF LC MS instrument with Agilent Dual Jet Stream Technology as ESI source (Agilent Technologies, USA). For LC an ACQUITY UPLC HSS T3 column (2.1 x 100 mm, 1.8 μm particle size, Waters Corporation, USA) was used at a temperature of 40°C and a flow rate of 0.5 ml min^-1^. The solvent system consists of solvent A (water/formic acid (100/0.1, *v/v*) and solvent B (acetonitrile/formic acid (100/0.1, *v/v*). The gradient was comparable as applied for UPLC TOF-MS analysis. The Q-TOF MS instrument was operated with a detection frequency of 2 GHz in the Extended Dynamic Range and the targeted MS/MS mode. The source conditions were: gas temperature: 250°C; drying gas flow: 8 l min^-1^; nebulizer pressure: 35 psi; sheath gas temperature: 300°C; sheath gas flow: 8 l min^-1^; VCap voltage: 3 kV; nozzle voltage: 200 V; fragmentor voltage: 100 V. Samples were ionized in negative and/or positive ESI mode with collision energy 10–30 eV. Isolation of precursor ions occurred within the narrow isolation width of 1.3 *m/z*. Data were acquired by Mass Hunter Workstation Acquisition software B.05.01 (Agilent Technologies, USA). Mass Hunter Qualitative Analysis B.06.01 (Agilent Technologies, USA) was used for data analysis.

### Synthesis of N-acetyl-amino adipate

Synthesis of N-acetyl-amino adipate was performed according to [29]. 1 g of α-amino adipate (Sigma, Germany) was dissolved in 5 ml of 2 M NaOH. 2.5 ml acetic anhydride was added, mixed and incubated for 20 min on ice. The reaction mixture was transferred to a column with a strong cation exchange resin (Serva Electrophoresis, Germany) and washed with 300 ml water. Effluent and washings were combined and evaporated under reduced pressure in a rotary evaporator. The presence of the product N-acetyl-amino adipate was verified by exact mass (203.086 Da) and MS/MS fragmentation studies.

## Results

### CYP94B1, CYP94B2, CYP94B3, and CYP94C1 regulate the sequential oxidation of JA-Ile to 12-hydroxy-JA-Ile and 12-carboxy-JA-Ile semi-redundantly

To compare the catalytic function of the different CYP94-enzymes we first analyzed the jasmonate profiles of the different loss-of-function mutants after mechanical wounding of the leaves. For this purpose, the different plant lines including Col-0 as control were grown under short day conditions and the rosette leaves of six-week-old plants were wounded three times across the mid vein and harvested 0.5 h, 1 h, 2 h and 5 h after wounding (hpw). The amounts of JA, JA-Ile, 12-hydroxy-JA-Ile, 12-carboxy-JA-Ile, and 12-hydroxy-JA were quantified by LC/MS-MS analysis.

**Fig 2:**
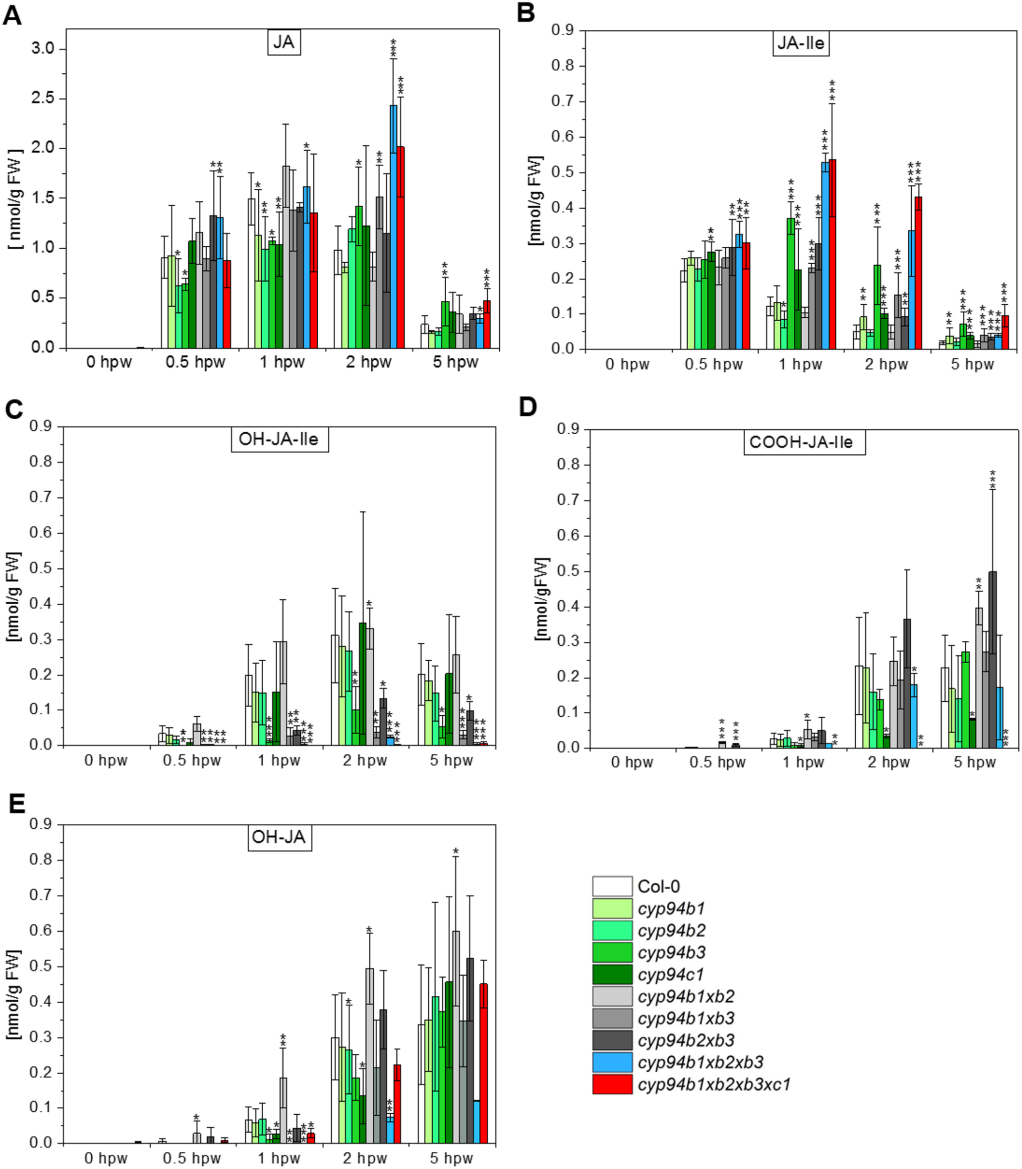
Jasmonate profiles of *CYP94*-mutants after wounding. Plants were grown under short day conditions (8 h light / 16 h dark) at 22°C. Rosette leaves of six-week-old plants were wounded three times across the mid vein. Damaged rosette leaves were harvested for phytohormone extraction at indicated time points according to hours post wounding (hpw). Extracts of pooled rosette leaves were analyzed by LC-MS/MS. Quantitative data of the wounding time course are given in nmol/g FW for A) jasmonic acid (JA), B) jasmonic acid-isoleucine (JA-Ile), C) 12-hydroxy-jasmonic acid-isoleucine (OH-JA-Ile), D) 12-carboxy-jasmonic acid-isoleucine (COOH-JA-Ile) and E) 12-hydroxy-jasmonic acid (OH-JA). Each data point represents the mean value ± SD of four biological replicates. Asterisks indicate significant differences between Col-0 and mutant according to student’s t-test (* p ≤ 0.05, ** p ≤ 0.01, *** p ≤ 0.001). For a detailed one-way analysis of variance (using the Tukey post-hoc test, *p*<0.05) we refer to S2 Table.

As shown in Fig 2A, we observed a constant increase of JA in all investigated plant lines from 0.5–2 hpw followed by a decrease at 5 hpw. For the early time-points (0.5 hpw and 1 hpw) we found only minor differences of JA accumulation. At 2 hpw, however, a significantly increased JA content (2.0–2.5-fold) was detected for the triple mutant and the quadruple mutant compared to Col-0 (see S2 Table for detailed ANOVA-analysis). These differences in the JA levels persisted and were also visible at 5 hpw suggesting an at least delayed degradation of JA.

The levels of JA-Ile differed only slightly at 0.5 hpw when the mutant plants were compared to Col-0 (Fig 2B). At the following time points, however, the amount of JA-Ile was increased significantly in all mutant plants with an impaired CYP94B3- and CYP94C1-functionality (*cyp94b3*, *cyp94c1*, *cyp94b1xcyp94b3* and *cyp94b2xcyp94b3*, the triple and quadruple mutant, see S2 Table for detailed ANOVA-analysis). Especially the triple and quadruple mutant plants showed JA-Ile levels that were increased by a factor of 4–9 compared to Col-0. Again, the relative differences persisted and were also visible at 5 hpw suggesting an at least delayed degradation of the active wound signal.

12-hydroxy-JA-Ile represents the first (intermediary) formed product in the oxidative deactivation of JA-Ile. Consequently, its accumulation is shifted to later time-points compared to JA-Ile and reaches its maximum in Col-0 at 2 hpw (Fig 2C). The amount and profile of 12-hydroxy-JA-Ile accumulation appeared similar for Col-0, the *cyp94b1*-, *cyp94b2*- and *cyp94c1* single- as well as for the *cyp94b1xcyp94b2* double loss-of-function mutants. In contrast, the CYP94B3 related single (*cyp94b3*) and double mutants (*cyp94b1xcyp94b3 and cyp94b2xcyp94b3*) as well as the triple and quadruple mutant showed highly reduced 12-hydroxy-JA-Ile content compared to Col-0 in all samples analyzed (see S2 Table for detailed ANOVA-analysis). Whereas triple mutants showed low amounts of 12-hydroxy-JA-Ile at 2 hpw, exclusively, the quadruple mutant is the only plant line in which 12-hydoxy-JA levels were below the detection limit.

A further compound in the oxidative inactivation pathway of JA-Ile is 12-carboxy-JA-Ile, which results from an additional four electron oxidation of 12-hydroxy-JA-Ile. Accordingly, the wound-induced accumulation of this compound is shifted to even later time-points compared to its precursors JA-Ile and 12-hydroxy-JA-Ile (Fig 2D). While the mutant plants with an impaired CYP94B functionality (*cyp94b1-*, *cyp94b2-* and *cyp94b3* single as well as the different double and triple loss-of-function mutants) exhibited an accumulation profile similar to that of Col-0, the amount of 12-carboxy-JA-Ile was highly reduced in the *cyp94c1* single as well as in the quadruple mutant at every time-point we analyzed (see S2 Table for detailed ANOVA-analysis). In the latter plant line, the amount of this compound was even below the detection limit.

Recently, 12-hydroxy-JA has been identified as another inactive jasmonate derivative that can be formed through deconjugation of 12-hydroxy-JA-Ile potentially by the back reaction of the amido hydrolases IAR3 and ILL6 [6]. Interestingly, we found that the profile of 12-hydroxy-JA accumulation was similar for most of the plant lines investigated (Fig 2E). Differences in the amount of this compound compared to Col-0 were detected for the *cyp94b1xcyp94b2* mutant (2-fold increased) as well as for the *cyp94b1xcyp94b3* double and the triple mutant (2-3-fold decreased) at 1 hpw. This trend persisted over the here analyzed time course.

### CYP94 functionalities regulate flowering time and JA-response in roots but are not essential for correct flower development

In order to investigate cooperative effects of the different *CYP94*-genes on plant physiology and plant development, we compared the visible phenotypes of the different loss-of-function mutants to Col-0. Leaf shape (S2A Fig), leaf area and rosette appearance did not differ between the different six-week-old plants (S2B Fig). In addition, for none of the plants severe stunting or color-variance could be observed.

JA-deficient mutant plants suffer from male-sterility as they are known i) to form non-viable pollen, ii) to exhibit a reduced filament elongation and iii) to show a delayed dehiscence of anthers [30, 31]. However, it is yet not entirely clear how jasmonate homeostasis is influencing the time-point of flowering. In order to tackle this question, we determined the flowering time of the different plant mutant lines by counting the leaves at an inflorescence length of 1 cm. As shown in S3 Fig, Col-0 started to flower after the development of 11 rosette leaves on average; this number varied for the different plant lines: Of the single T-DNA insertion lines, *cyp94b3* and *cyp94c1* showed the strongest effect. Plants with a *CYP94B3* T-DNA insertion started to flower with a higher number of leaves compared to WT. When in addition to CYP94B3 CYP94B1 was defunctionalized (*cyp94b1xcyp94b3*) the time-point of flowering was even more delayed. This plant line started to flower after the development of 13 leaves. On the other hand the CYP94C1 mutant started to flower at an earlier time-point than the WT. Interestingly, this effect was enhanced by the additional defunctionalization of all three CYP94B-enzymes. The respective quadruple mutant started to flower already after the development of 9 rosette leaves. This finding suggests that CYP94B3 and CYP94C1 might function as antagonistic regulators controlling flowering-time.

Several studies demonstrated that jasmonates also function as key regulators during flower development and thus also affect flower morphology [32]. We therefore examined next whether an impaired jasmonate catabolism caused by the loss of cyp94 functionality leads to alterations in flower morphology. As shown in S4 Fig, we found for all the different plants lines investigated here a number of petals, sepals and stamen that were according to the typical flower symmetry of Brassicaceae [33]. Thus, as Col-0 all the different mutant plant lines were fertile and reached a full life cycle.

**Fig 3:**
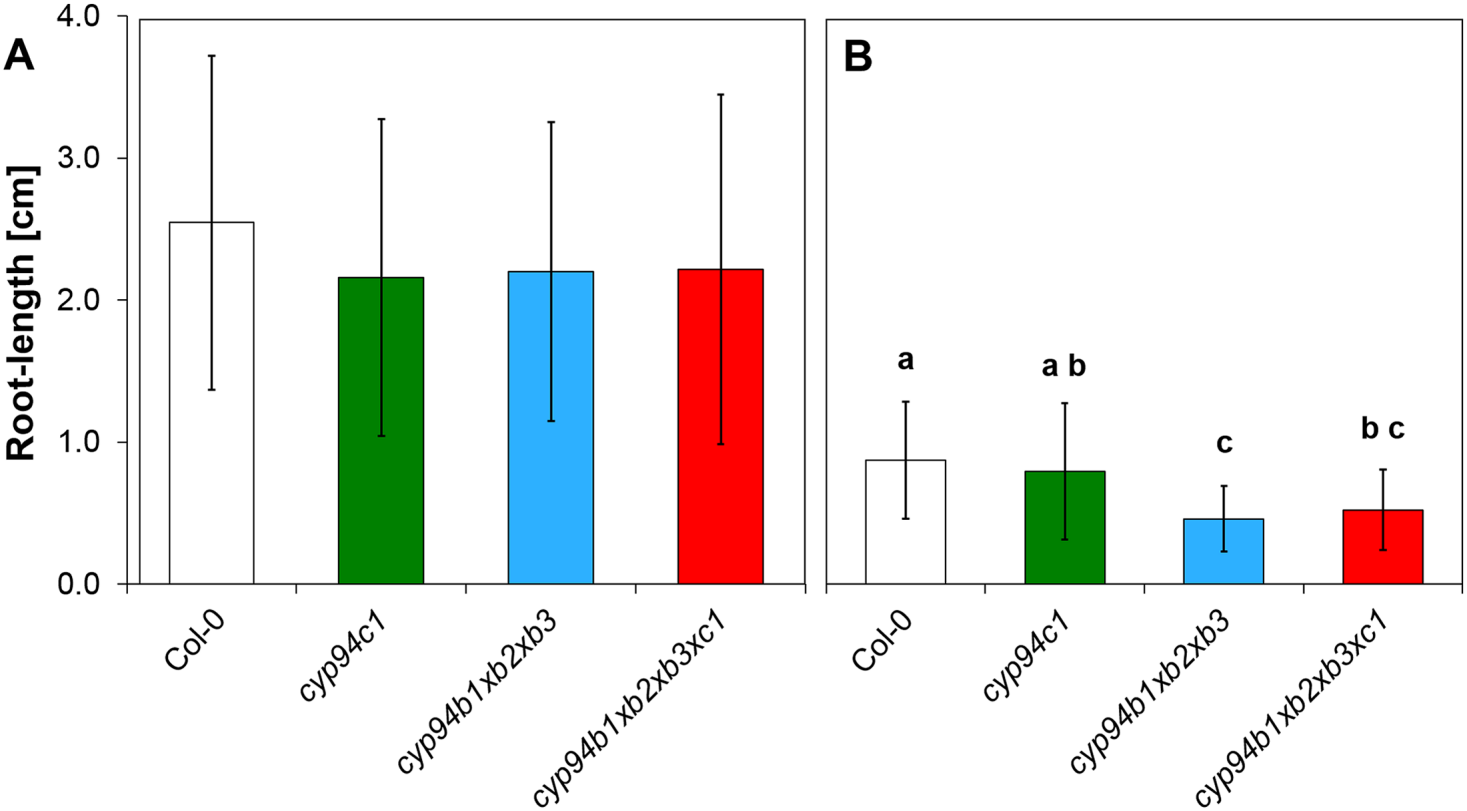
Root-growth assay of Col-0, *cyp94c1*, *cyp94b1xb2xb3* and *cyp94b1xb2xb3xc1* in the absence and presence of jasmonic acid (JA). Root-length of 10 d old seedlings grown under continuous light conditions was measured in **A**) absence and **B**) presence 10 μM JA. Each data point represents the mean value of 3 independent experiments with 10 seeds per plant line. Letters indicate whether the respective mean values are significantly different as determined by the analysis of variance employing the Tukey post-hoc test.

Next, we aimed to examine the effect of an impaired CYP94 functionality towards JA-application. The sensitivity of plant lines towards JA was analyzed by a root-growth assay. Since our results shown above as well as different studies of others ([12, 15]) indicated that CYP94B-enzymes (in particular CYP94B1 and CYP94B3) and CYP94C1 are responsible for the catalysis of the different reactions in the oxidative jasmonate deactivation, we concentrated in this analysis on the *cyp94c1* mutant, the triple and the quadruple mutant. The control treatment without exogenous JA shows no differences in the root length of the mutants and the Col-0 (Fig 3A). Interestingly with JA application, the triple and the quadruple mutants both showed a two-fold reduction in the root length whereas the *cyp94c1* mutant was apparently unaffected and displayed a similar root length as Col-0 (Fig 3B). These findings revealed an elevated sensitivity of the CYP94B-triple and the quadruple mutant towards JA and suggested the importance of CYP94B-enzymes in the termination of JA-signals.

### Expression of CYP94-genes is tissue specific and partly overlapping

In order to investigate the expression level of the different *CYP94* coding genes in different plant tissues (roots, shoots, leaves, petioles, flowers and siliques), we performed a semi-quantitative RT-PCR analysis (S5 Fig). In the root tissue the expression of *CYP94B1* was found to be lower than the expression of the different other *CYP94*-genes investigated. On the other hand, this gene was expressed in the shoots as well as *CYP94B3* and *CYP94C1* at similar levels whereas no expression was found for *CYP94B2*. In leaf tissue, only very weak expression levels could be detected for *CYP94B1* and *CYP94B2*. The genes coding for *CYP94B3* and *CYP94C1* were found to be expressed to a similar extend. In the petiole *CYP94C1* is the only gene that is strongly expressed to a higher extend; for the other genes, only very low expression levels were observed. In contrast to that, in floral tissue the strongest expression was found for the *CYP94B1* and *CYP94C1* coding genes while *CYP94B2* and *CYP94B3* were only expressed to a lower amount. The expression profile of *CYP94* coding genes in the siliques was similar to that of shoots with the exception of *CYP94B1* that was only expressed in moderate amounts in this tissue. As a result, we found that *CYP94B1* is mainly expressed in the shoots and flowers whereas *CYP94B2* shows its strongest expression in the roots and is also expressed in the flowers albeit to a lower extend. The expression of *CYP94B3* was detected in every tissue with varying levels. In the petiole and in the flower, the expression was less intensive compared to other tissues. Notably, the only gene that was almost equally expressed in the different plant organs was encoding for *CYP94C1*.

In order to gather more information on the expression of the *CYP94* encoding genes in *A*. *thaliana*, we next performed an expression analysis in the vegetative and reproductive organs by employing the β-Glucoronidase reporter system. In S6 Fig pictures of the reproductive organs (inflorescence, flower, silique) of the different *CYP94*-promoter:GUS-lines are shown. Except for the *CYP94B2*-promoter:GUS-plants all other investigated plant lines exhibited a distinct blue staining of the reproductive organs; here, the vasculature showed an intensive blue color. Above all the *CYP94B1*-promoter:GUS constructs showed a clear GUS activity in the flowers. Also the vasculature of sepals, petals and stamen was intensively stained blue. The GUS activity was stronger in the vasculature of the stamen than in other floral tissues of the *CYP94B3*-promoter:GUS-lines. The respective *CYP94B3* lines also displayed a strong GUS-activity in the vasculature of petals and stamen but the signal was notably not detectable in the sepals. For those later constructs, the GUS-activity of the siliques was mainly localized to the basal part of the silique but spread into the vasculature of the whole silique.

**Fig 4:**
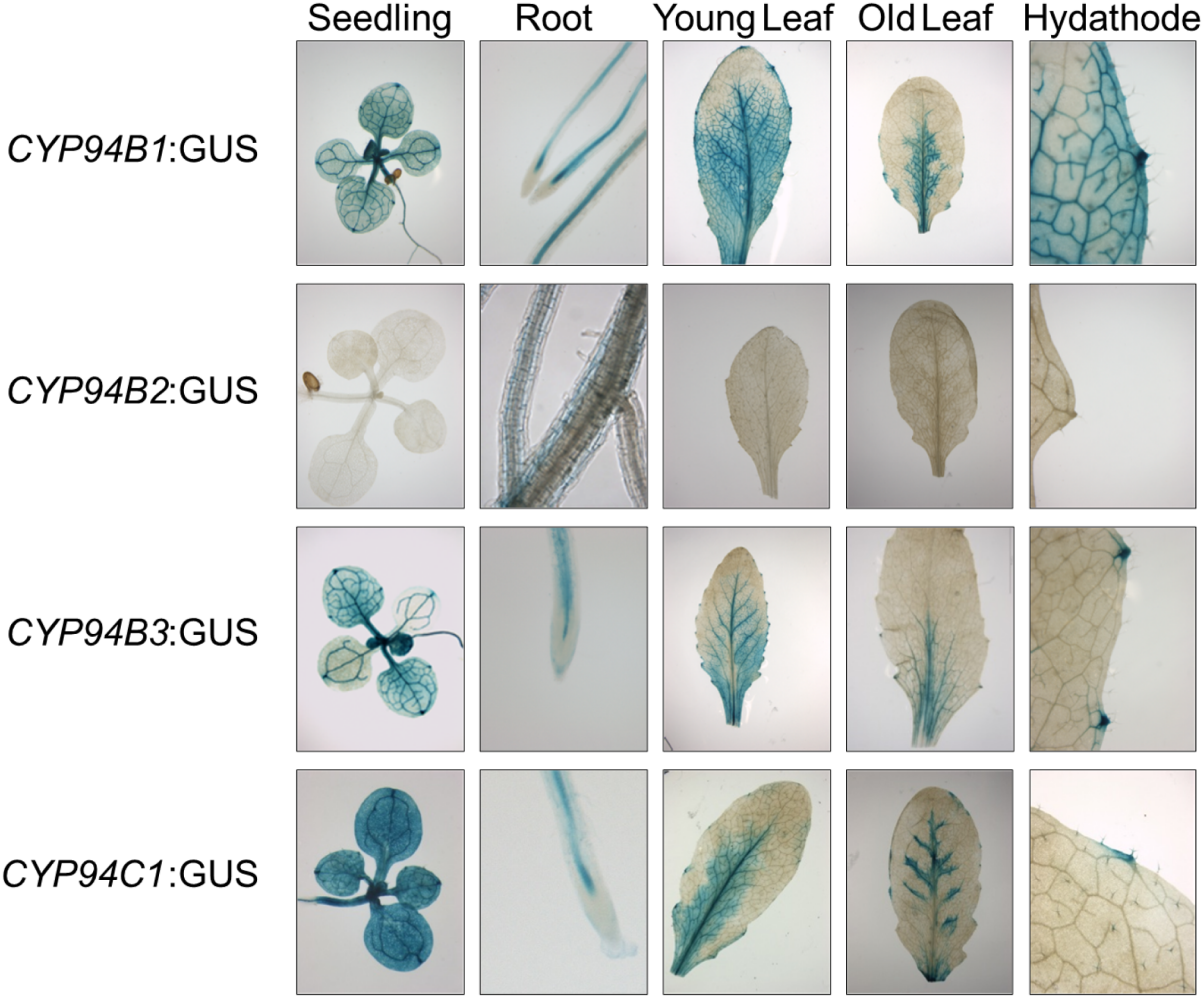
Expression profile of promoter:GUS-constructs for CYP94B1, CYP94B2, CYP94B3 and CYP94C1 in vegetative organs of *A*. *thaliana*. Transformed plants were grown on soil under long-day (16 h light / 8 h dark) conditions. Seedlings were grown on ½ MS plates. All plant lines were stained with 2 mM X-Gluc. Staining was performed with two independent plant lines per construct with comparable results. Staining was performed ≥3 times with each line with comparable results.

Pictures of the vegetative organs (seedling, root, young leaf, old leaf, hydathode) of the different *CYP94*-promoter:GUS-lines are shown Fig 4. The vasculatures of leaves and roots of *CYP94B1*-promoter:GUS-lines were intensively stained blue suggesting a strong expression of *CYP94B1* in these tissues. The only vegetative organ of *CYP94B2*-promoter:GUSplant lines where a very weak blue staining could be observed were the roots. In contrast to the other investigated plant lines the staining was not restricted to the vasculatures but rather to the surrounding endodermis and root cortex. Interestingly, we found for the expression of *CYP94B3*-promoter:GUS and for the respective *CYP94C1*-promoter:GUS constructs a similar pattern as for *CYP94B1*. Again the vasculature of the leaves and roots were intensively stained, whereas the root tip was again unstained. Hydathodes were intensively stained in all ages of the leaves; however, with an increasing age the staining got more restricted to the midrib of the leaf.

### Identification of 12-carboxy-JA as a novel jasmonate catabolite in *A*. *thaliana* flowers

**Fig 5:**
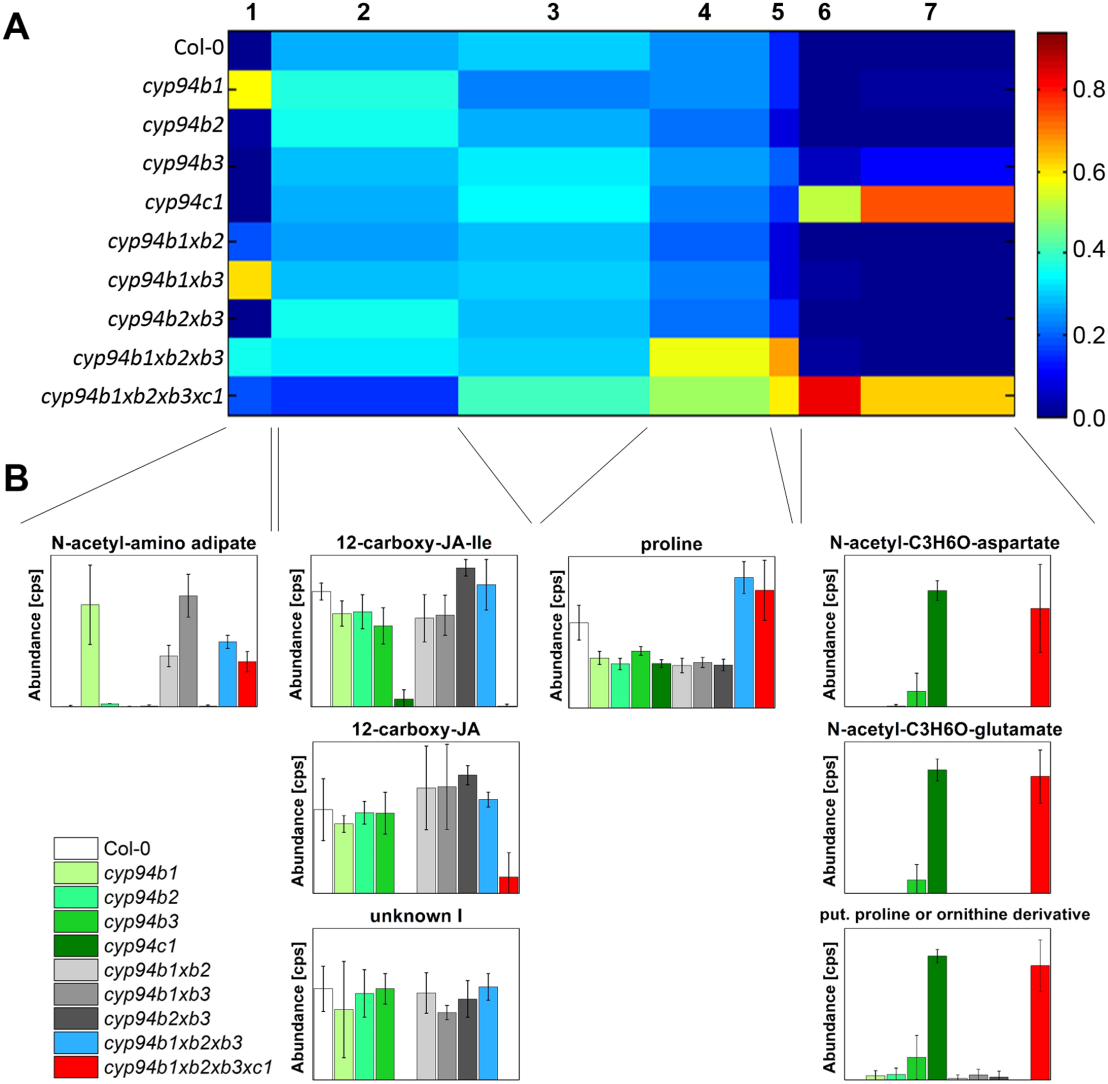
Metabolite fingerprinting analysis of flowers of Col-0 and *CYP94*-mutant lines. Col-0 and *cyp94b1*, *cyp94b2*, *cyp94b3*, *cyp94c1* single, double, triple and quadruple mutant plants were grown under long day conditions (16 h light/8 h dark) at 22°C. Flowers were harvested at stage 13–14, homogenized, extracted by two-phase-extraction and analyzed by UHPLC/ESI-TOF MS. A subset of 164 high quality metabolite features (FDR < 10^−4^) derived from the positive ionization mode of the polar and the non-polar extraction phase were obtained. **A**) For metabolite-based clustering by means of one-dimensional self-organizing map (1D-SOM) 7 clusters were selected. The width of a cluster is proportional to the number of features assigned to the cluster. The heat map colors represent average intensity values (see color map right-hand side). For analysis two independent experiments with at least two pools of flowers each were used. Reliable features of both experiments were used for 1D-SOM representation. **B**) Relative amount of selected metabolite markers. Identities of the markers were confirmed by MS^2^ analysis. Data analysis and visualization were performed with MarVis [25].

**Fig 6:**
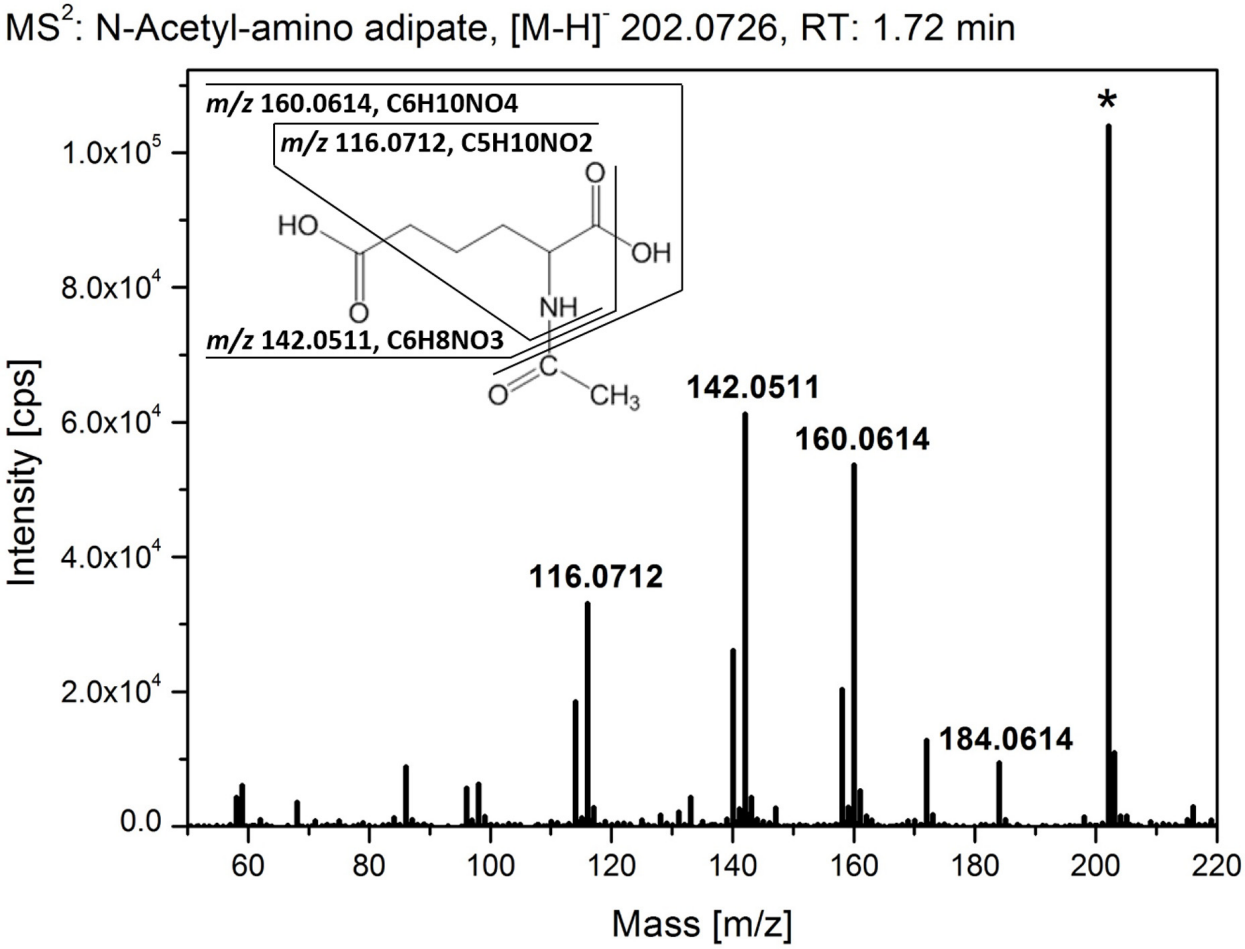
High resolution MS^2^ analysis of N-acetyl-amino adipate. UHPLC-ESI QTOF-MS fragmentation analysis of a metabolite marker accumulating in flowers with impaired CYP94B1 functionality. MS^2^ spectrum of N-acetyl-amino adipate (*m/z* 202.0726, retention time 1.72 min) is shown for the negative ionization mode with a collision energy of 10 eV. Loss of the N-bound acetyl group leads to the fragment of *m/z* 160.0614. Subsequent losses of water and the carboxy-group of the amino adipate result in the fragments of *m/z* 142.0511 and *m/z* 106.0614.

**Fig 7:**
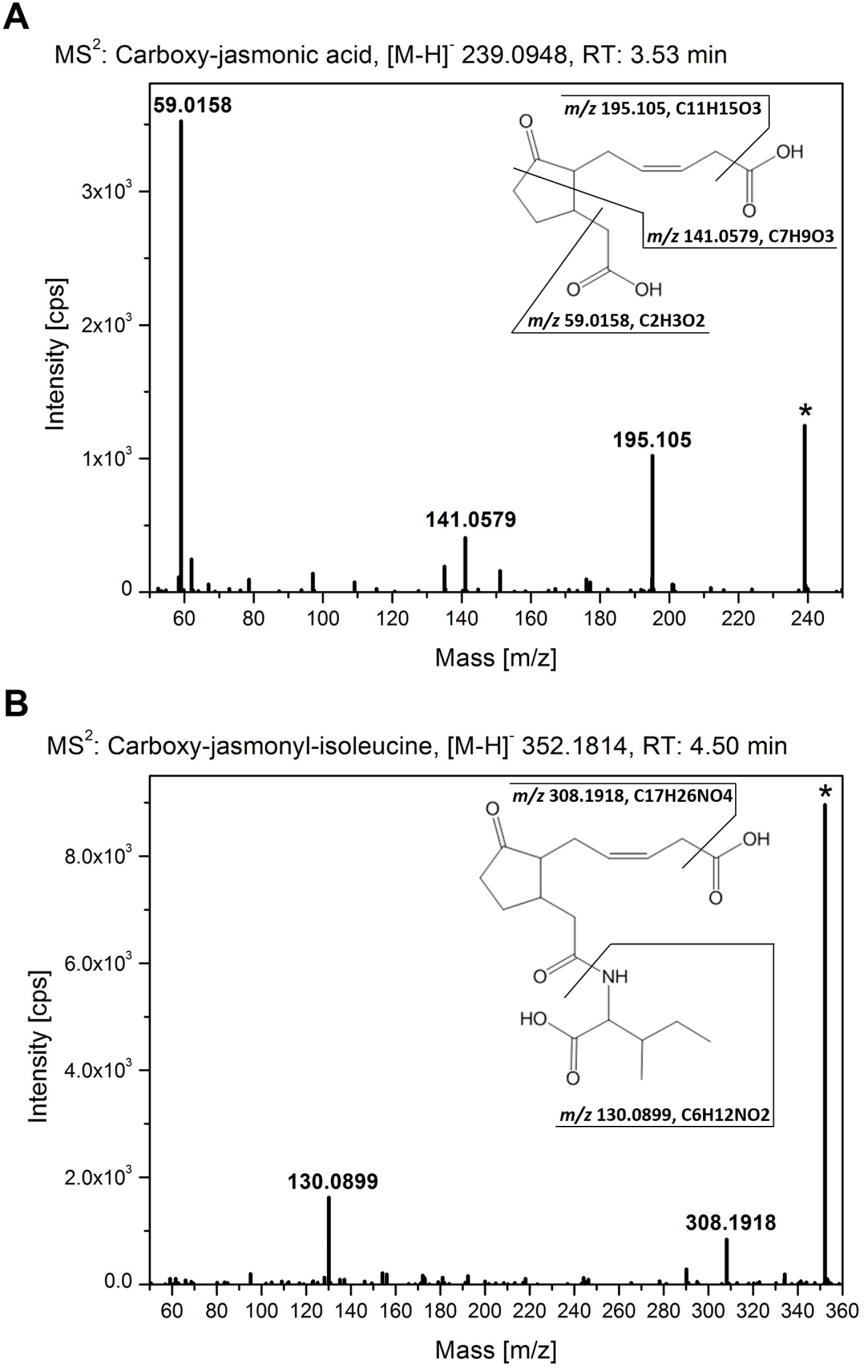
High resolution MS^2^ analyses of 12-carboxy-JA and 12-carboxy-JA-Ile. UHPLC-ESI QTOF-MS fragmentation analyses of metabolite markers depleted in flowers with impaired CYP94C1 functionality. MS^2^ spectra of **A)** 12-carboxy-JA (*m/z* 239.0948, RT 3.53 min) and **B)** 12-carboxy-JA-Ile (*m/z* 352.1814, RT 4.50 min) are shown for negative ionization mode with a collision energy of 10 eV. The loss of the ω-carboxy group leads to the fragments of *m/z* 195.1051 for 12-carboxy-JA and *m/z* 308.1918 for 12-carboxy-JA-Ile. The free α-carboxy group of 12-carboxy-JA results in the fragment of *m/z* 59.0159, while 12-carboxy-JA-Ile shows the fragment of *m/z* 130.0899 for isoleucine.

As illustrated in S5 and S6 Figs and shown by [14], most of the *CYP94*-genes are expressed in flowers of *A*. *thaliana* suggesting the presence of a diverse set of jasmonate derivatives that may orchestrate flower development [1]. The strongest transcript accumulation in flowers was shown for the *CYP94B1* and *CYP94C1*. In order to identify metabolic changes as consequence of the inactivation of the respective *CYP94*-genes, a non-targeted metabolite fingerprinting approach of Arabidopsis flowers (stage 12–14) was performed. For this purpose, the polar and non-polar extraction phases of flowers were analyzed. A set of 164 high quality features with a false discovery rate (FDR) < 10^−4^ was obtained (S3 Table). The features were grouped according to their relative intensity profiles within 7 clusters by means of one-dimensional self-organizing maps (1D-SOMs) using the toolbox MarVis [25]. The 1D-SOM representation allows a global overview of the metabolic changes of all mutant lines in respect to wild type (Fig 5A). Cluster 1 contains features, which show accumulation in plants with an impaired CYP94B1 functionality. The main metabolite represented by cluster 1 had the accurate mass of [M+H]^+^ 204.0849 from which the sum formula of C_8_H_13_NO_5_ could be deduced. Structure information obtained by MS^2^ fragmentation analyses by UHPLC-QTOF-MS allowed the assignment as N-acetyl-amino adipate (Fig 5B). The identity of this acetylated C6 amino dicarboxylic acid as a CYP94B1-specific marker could be unequivocally confirmed by comparison of the fragmentation pattern (Fig 6, Table 3) and retention time with that of the authentic standard, which was obtained by chemical synthesis. The clusters 2 and 3 of the 1D-SOM represent 79 features of rather indifferent intensity pattern. Surprisingly, there were three features included ([M+H]^+^ 241.1014, [M+H]^+^ 354.1898, [M+H]^+^ 443.1794) which showed accumulation only in plant lines with an intact CYP94C1 functionality (Fig 5B). In the *cyp94c1* single as well as in the quadruple mutant these features were absent or nearly absent. MS^2^ fragmentation analyses revealed the chemical structure of the first two features as the jasmonate derived compounds 12-carboxy-JA-Ile and 12-carboxy-JA (Fig 5B, Table 3). The identity of the new jasmonate 12-carboxy-JA was supported by comparison of its high resolution MS^2^ spectra with that of 12-carboxy-JA-Ile (Fig 7). Both ω-carboxy-jasmonates show a loss of the ω-carboxy group in the negative ionization mode, which led to the characteristic fragments of *m/z* 308.1918 for 12-carboxy-JA-Ile and of *m/z* 195.1051 for 12-carboxy-JA. Regarding 12-carboxy-JA, the free α-carboxy group results in the typical JA fragment of *m/z* 59.0159 (CH_3_-COO^-^), while the conjugation of Ile to the α-carboxy group in 12-carboxy-JA-Ile disables the formation of *m/z* 59.0159. Beside those carboxy-jasmonate derivatives, no further known jasmonates could be detected in the flowers by metabolite fingerprinting. Features with the reverse pattern to the carboxy-jasmonates are represented by clusters 6 and 7. The intensity profiles of these features show a strong enrichment in flowers of the *cyp94c1* single as well as in the quadruple mutant lines. Two of the compounds could be tentatively identified as an acetylated aspartate derivative ([M+H]^+^ 234.0958, C_9_H_15_NO_6_) and an acetylated glutamate derivative ([M+H]^+^ 248.1116, C_10_H_17_NO_6_) based on their high resolution MS^2^ spectra in the positive as well as in the negative ionization mode (Fig 5B, Table 3). In accordance with the accurate mass and the fragmentation pattern, both C4 and C5 amino dicarboxylic acids (Asp and Glu) seem to be N-acetylated and to harbor an additional C_3_H_6_O-unit. Clusters 4 and 5 contained 31 features that were enriched in the triple and quadruple mutant. Among those features, we found one that was identified as proline ([M+H]^+^ 116.0696). In conclusion, the strong expression of *CYP94B1* and *CYP94C1* in flowers agrees well with the remarkable chemotypes we describe here for the first time in mutants with impaired CYP94B1 functionality (accumulation of acetylated C6 amino dicarboxylic acid) as well as CYP94C1 functionality (accumulation of acetylated C4/C5 amino dicarboxylic acid derivatives) by metabolite fingerprinting analysis.

**Table 3:** Metabolite markers of *A*. *thaliana* flowers (stage13-14) as identified by metabolite fingerprinting and verified by MS^2^ analysis or coelution.

Metabolite marker	RT (min)	m/z	Detected ion	Calculated mass (Da)	Sum formula	Error (mDa)	Extraction phase	Cluster nr.	Compound identified by	MS^2^ fragmentation	CE (eV)
**Accumulated in *cyp94b1***											
N-Acetyl-amino adipate	1.72	204.0849	[M+H]^+^	203.0794	C8H13NO5	-1.8	polar	1	B: Metlin ID 3271 (Aminoadipate*), C	202.0726 [M-H]^-^, 184.0614 [M-H2O-H]^-^, 172.0619, 160.0616 [M-acetyl-H]^-^*, 158.0821, 142.0511 [M-acetyl-H2O-H]^-^*, 140.0717, 116.0712 [M-acetyl-CO2-H]^-^ *, 114.092*	10
**Depleted in *cyp94c1***											
COOH-JA	3.53	241.1014	[M+H]^+^	240.0998	C12H16O5	-5.7	polar	2	B: Metlin ID 3345 (Jasmonic acid)	239.0948 [M-H]^-^, 195.1051 [M-CO2-H]^-^, 59.0159	10
COOH-JA-Ile	4.50	354.1898	[M+H]^+^	353.1838	C18H27NO6	-1.3	polar	2	A [34]	352.1814 [M-H]^-^, 308.1918 [M-CO2-H]^-^, 130.0899 [Ile-H]^-^	10
Unknown I	2.08	443.1794	[M+H]^+^				polar	2	D		
**Accumulated in triple and quadruple mutant**											
Proline	0.52	116.0696	[M+H]^+^	115.0633	C5H9NO2	-1	polar	4	A: Metlin ID 29	116.0696 [M+H]^+^, 70.0658 [M-CH2O2+H]^+^	10
**Accumulated in *cyp94c1***											
N-Acetyl-C3H6O-aspartate (Asp-C5H8O2)	1.54	234.0958	[M+H]^+^	233.0899	C9H15NO6	-1.4	polar	7	B: Metlin ID 15 (Aspartate), ID 3769 (N-Acetyl-aspartate*)	234.0987 [M+H]^+^, 216.0865 [M-H2O+H]^+^, 198.0767 [M-2xH2O+H]^+^, 170.0808 [M-2xH2O-CO+H]^+^, 134.045 [Asp+H]^+^, 116.034 [Asp-H2O+H]^+^, 88.0397 [Asp-H2O-CO+H]^+^, 55.0547	10
			[M-H]^-^							232.084 [M-H]^-^, 214.0118 [M-H2O-H]^-^, 174.0427 [Acetyl-aspartate-H]^-^*, 132.0306 [Asp-H]^-^*, 130.0512 *, 115.0043 [Asp-NH2-H]^-^*, 88.0412 [Asp-H2O-CH2O-H]^-^*, 71.0139*, 58.0305*	10
N-Acetyl-C3H6O-glutamate (Glu-C5H8O2)	1.98	248.1116	[M+H]^+^	247.1056	C10H17NO6	-1.3	polar	7	B: Metlin ID 19 (Glutamate), ID 3325 (N-Acetyl-glutamate*)	248.1137 [M+H]^+^, 230.1020 [M-H2O+H]^+^, 212.091 [M-2xH2O+H]^+^, 184.0968 [M-2xH2O-CO+H]^+^, 148.0606 [Glu+H]^+^*, 130.0497 [Glu-H2O+H]^+^*, 102.0554 [Glu-CO2+H]^+^*, 84.0447*, 83.0497, 55.0549	12
			[M-H]-							246.0989 [M-H]^-^, 228.0894 [M-H2O-H]^-^, 188.0583 [Acetyl-glutamate-H]^-^*, 170.0468 [Acetyl-glutamate-H2O-H]^-^*, 146.0466 [Glu-H]^-^*, 144.0688*, 128.0359 [Glu-H2O+H]^+^*, 117.056, 102.0568 [Glu-CO2-H]^-^*, 59.0155*	14
Put. Proline or Ornithine derivative	1.83	319.0773	[M+H]^+^	*318*.*0699*	*C11H14N2O9*	0.1	polar	7	B: Metlin ID 29 (Proline*), ID 45121 (Ornithine*)	319.0799 [M+H]^+^, 128.0702, 116.0709*, 70.0651*	
	0.60	319.0776	[M+H]^+^	*318*.*0699*	*C11H14N2O9*	0.4	non-polar	7			

A) MS^2^ fragment information from Glauser G, Grata E, Dubugnon L, Rudaz S, Farmer EE, Wolfender JL. Spatial and temporal dynamics of jasmonate synthesis and accumulation in Arabidopsis in response to wounding. J. Biol. Chem. 2008;283: 16400–7 or METLIN database (http://metlin.scripps.edu).

B) MS^2^ fragment information from authentic standard or METLIN database (http://metlin.scripps.edu) information of the corresponding non-conjugated compound (in brackets). In case only this partial structural information was available, the substance and the corresponding fragments were marked with *.

C) Coelution with authentic standard.

D) Exact mass measurement only.

## Discussion

Several recent studies reported a function of CYP94-enzymes in the oxidative inactivation pathway of JA-Ile. It has generally been established that in particular CYP94B1, CYP94B3 and CYP94C1 are involved in the stepwise ω-oxidation of JA-Ile (*cf*. Fig 1). Whereas CYP94B3 and CYP94B1 are mainly responsible for the two-electron oxidation of JA-Ile to 12-hydroxy-JA-Ile, CYP94C1 has its primary function in the sequential oxidation of this compound to 12-oxo-JA-Ile and finally to 12-carboxy-JA-Ile [12, 13, 35, 36]. However, recent studies employing *CYP94*-loss of function mutants demonstrated, that despite these catalytic preferences, CYP94-enzymes exhibit overlapping functions and might act redundantly in order to ensure functional jasmonate catabolism [14, 15]. In addition, it has been proposed that alternative routes for the signal inactivation may exist. To expand the knowledge on the role of CYP94-enzymes in this pathway, we performed a comprehensive analysis in which we characterized *cyp94b1*, *cyp94b2*, *cyp94b3* single, double and triple loss of function mutants, the *cyp94c1* single mutant, as well as a quadruple mutant that was deficient in the functionality of all four mentioned CYP94-enzymes. We started our study by analyzing and comparing the jasmonate profiles of different *CYP94*-mutants in leaves which had been stressed by a mechanical wounding stimulus. It has recently been demonstrated that in contrast to biotic stress resulting from pathogen infection, stress induced by mechanical leaf injury results in a transient and strong accumulation of mainly Ile-conjugated (ω-oxidized) jasmonate derivatives [14]. We observed a maximal accumulation of JA-Ile 30 min after wounding, while the maximal levels of ω-oxidized JA-Ile derivatives were reached at later time-points (Fig 2). Along the same line, we found that at early to middle time points of wounding (0.5–2 hpw) JA was the most abundant jasmonate while at the last time point (5 hpw) oxidized jasmonates were predominately present. A similar temporal jasmonate distribution has been reported recently [14] and reflects that ω-oxidation of JA-Ile occurs down-stream of its formation and is thus consistent with the idea of an oxidative inactivation pathway of the active compound JA-Ile [7, 12, 37].

### The levels of 12-hydroxy- and 12-carboxy-JA-Ile are regulated by the activity of CYP94B1, CYP94B2, CYP94B3, and CYP94C1

The important role of CYP94B3 for the oxidation of JA-Ile to 12-hydroxy-JA-lle has been reported recently [10–13]. In line with these studies, we also found that mutants lacking this functionality (*cyp94b3* single, double, triple and quadruple mutants) accumulate increased JA-Ile levels whereas the amount of the respective ω-oxidized derivative, 12-hydroxy-JA-Ile, is significantly reduced. In all the single *CYP94* loss of function mutants investigated here, the strongest effect on the formation of 12-hydroxy-JA-Ile was observed in *cyp94b3* exhibiting a three-fold reduction. As shown above and reported previously [12], however, we still observed formation of 12-hydroxy-JA-Ile by the other CYP94 enzymes reflecting the redundant activity of CYP94 enzymes [14]. Accordingly, we found that the amount of 12-hydroxy-JA-Ile was further reduced when additionally to CYP94B3 also CYP94B1 or CYP94C1 activity was missing, suggesting that all three enzymes also possess the capacity to hydroxylate JA-Ile. The fact that the amount of 12-hydoxy-JA-Ile was stronger reduced in *cyp94b3xcyp94b1* than in *cyp94b3xcyp94c1* suggests that cyp94b1 hydroxylates JA-Ile more efficiently than cyp94c1 as has also been reported before [15]. Interestingly, we observed, that in the triple mutant lacking all three CYP94B-enzymes formation of 12-hydroxy-JA-Ile was more severely reduced than in any of the investigated double mutants. This suggests that CYP94B2 is involved in hydroxylation of JA-Ile albeit to a minor extent. In the quadruple mutant lacking CYP94C1 in addition to all three CYP94B-enzymes, formation of 12-hydroxy-JA-Ile was nearly fully abolished. It is well known that besides its catalytic activity as 12-hydoxy-JA-Ile oxidase, CYP94C1 also has the capacity to hydroxylate JA-Ile [12, 13]. This explains the small amounts of 12-hydroxy-JA-Ile in the triple mutant and the nearly complete absence of this compound in the quadruple mutant. The main catalytic activity of CYP94C1 is, however, the sequential oxidation of 12-hydroxy-JA-Ile towards 12-oxo-JA-Ile and 12-carboxy-JA-Ile [13]. Our results are in-line with these studies as the loss of CYP94C1 functionality leads to a severe reduction of 12-carboxy-JA-Ile as shown by the analysis of the *CYP94C1* single and the quadruple mutant. Neither of the investigated *CYP94B* single, double or triple mutant(s) showed a reduction in the level of 12-carboxy-JA-Ile as strong as that observed in both the CYP94C1 related mutants. Here, on the other hand, a participation of the CYP94B in the oxidation of 12-hydroxy-JA-Ile to 12-carboxy-JA-Ile is indicated as only the quadruple mutant completely lacks this highly oxidized derivative.

### Jasmonate profiles of different CYP94 mutants suggest direct hydroxylation of unconjugated JA as an additional route of JA catabolism

As shown in Fig 2 JA accumulation was significantly increased at 2 hpw in mutants lacking all three CYP94B functionalities (triple and quadruple mutant plants) but not in the single or double mutants. For the triple mutant this JA-increase was accompanied by substantially decreased level of 12-hydroxy-JA. On the one hand, this finding might be explained by the fact that due to the lack of CYP94B-enyzmes, JA-Ile cannot be further metabolized and thus the accumulating amounts of this compound lead to a tailback accumulation of JA. On the other hand, this finding might also indicate that CYP94B-enzymes are involved in the conversion of JA to 12-hydroxy-JA. Nevertheless, it was reported for CYP94B3 (as well as for CYP94C1) that the heterologously expressed enzyme(s) cannot oxidize JA [11, 12]. Further support for the hypothesis of a direct hydroxylation of JA towards 12-hydroxy-JA comes from the following observation: As discussed above, we found that the quadruple mutant is nearly fully deficient in the formation of 12-hydroxy-JA-Ile. Still, this mutant was capable of forming unconjugated 12-hydroxy-JA in significant amounts as in the Col-0. Consequently, this high metabolite level cannot solely derive from the deconjugation of the respective 12-hydroxy-Ile-conjugate from a nearly depleted substrate pool (*cf*. Fig 2).

It is known that 12-hydroxy-JA can also be formed by deconjugation of 12-hydroxy-JA-Ile catalyzed by the two JA amido hydrolases ILL6 and IAR3 [6, 7]. Expression of those genes is up-regulated upon stress by increased levels of JA. It has been proposed that under those conditions ILL6 and IAR3 are responsible for the hydrolysis of excess JA-Ile that may cause harmful effects to the plant [6]. Consequently, the hyper-accumulation of JA and JA-Ile (2 hpw) in the triple and quadruple mutant might lead to an increased expression of amido hydrolases that in turn reduce JA-Ile levels by deconjugation and thus increase the levels of JA.

In this context, it is important to note that Koo and co-workers observed that an *iar3xill6* mutant, deficient in both amido hydrolases, still produced 12-hydroxy-JA levels which were reduced only by a factor of two compared to those of Col-0. From that finding the authors hypothesized that 12-hydroxy-JA might be formed *via* an unknown pathway and speculated on the direct hydroxylation of JA by a so far unidentified P450 [6]. Such an enzyme has been identified in *Magnaporthe oryzae* recently [38]. Our results from the analysis of the different *cyp94b* and *cyp94c1* single and double loss of function mutants did not allow to assign which CYP94-enzyme might be responsible for the ω-hydroxylation of JA. In fact, our data rather suggest that all three CYP94B-enzymes as well as CYP94C1 do not necessarily catalyze this reaction.

### CYP94B-enzymes control JA-response in Arabidopsis roots

It is known that exogenous application of JA-Ile inhibits root growth and it has been demonstrated that Arabidopsis plants deficient in the two CYP94 enzymes CYP94B3 and CYP94C1 are more sensitive to JA-Ile than Col-0 [11, 12]. As discussed above we found a major influence of CYP94B1, CYP94B3 and CYP94C1 on the oxidative inactivation of JA (*cf*. Fig 2). In order to further evaluate the *in vivo* function of these enzymes and to investigate their impact on jasmonate sensitivity we challenged the roots of the *cyp94c1* single mutant plant as well as of the triple and quadruple mutant plants with exogenous JA and analyzed the effect on the root length. Similar to what was reported before [12] we also found that the single *cyp94c1* mutation has no significant influence on the root length (Fig 3). However, in case of the triple mutant that lacks the three CYP94B-enzymes, severe effects were observed upon JA treatment suggesting that this mutant exhibited an increased JA-sensitivity. The quadruple mutant, which is deficient in all three CYP94B and the CYP94C1-enzyme, showed a similar behavior suggesting that the additional loss of CYP94C1 activity did not further affect JA-sensitivity in roots. This finding suggests that mainly CYP94B-enzymes contribute to JA-response in roots whereas CYP94C1 plays only a minor role. When the analogous experiment was performed with the *cyp94b1xcyp94b3* and the *cyp94b1xcyp94b3xcyp94c1* mutants, no differences in the root length could be observed, as recently reported by Poudle and co-workers [17]. Since the plant lines used in this study still exhibited intact *CYP94B2*, the different results might be attributed to the functionality of this gene. A potential function of the CYP94-enzymes for root-growth is also reflected by our expression analyses. Here we found that indeed all four enzymes are expressed in significant amounts in the roots (S5 Fig).

In a recent study Widemann and co-workers showed that upon flower opening (stage 13–14) expression of *CYP94B1*, *CYP94B3* and *CYP94C1* is maximal and that of those enzymes *CYP94B3* showed the weakest expression [14]. In line with these results we found a similar expression pattern in flowers harvested at the mid-flowering stage 12–14 (S5 Fig). Promoter:GUS analysis further confirmed the partly overlapping spatial distribution of *CYP94*-expression in the floral parts of the plant: *CYP94B1* is mainly expressed in the vasculature of sepals, petals and stamen and in the upper part of the pistil, whereas expression of *CYP94C1* was only detected in the vasculature of stamen and petals but was absent in sepals. *CYP94B3* showed a very similar expression pattern as *CYP94B1* but seemed to be expressed stronger in the vasculature of the stamen compared to other floral tissues. These results suggest a partly overlapping but also tissue-specific expression for those genes [15]. We would like to note at this point that a more specific expression of CYP94B1 (*e*.*g*. in the stamen, petals and filaments), CYP94B3 (*e*.*g*. in the pistil) and CYP94C1 (*e*.*g*. in the anthers) has been observed before by other groups [14, 15]. Some differences in expression might be attributed to the varying lengths of the putative promoter regions that have been used for generation of the different GUS-constructs used in the respective studies.

### CYP94C1 functionality influences flowering time and is essential for the formation of 12-carboxy-JA, a novel JA-metabolite in Arabidopsis flowers

As noted above, roots and flowers were the only tissues in which we detected transcripts of every *CYP94*-gene investigated here in varying but sufficient amount by semi-quantitative RT-PCR. However, although the loss of CYP94-functionalities did not affect flower morphology (*cf*. S4 Fig) we found evidence that the temporal development of the flower is affected by these deletions (S3 Fig) and that especially CYP94B3 and CYP94C1 might play an important role in controlling flowering time. This finding was surprising as a recent study demonstrated that the flowering-time is essentially not influenced when the AOS-dependent biosynthesis of JA was disrupted [39]. However, we hypothesized from our two observations that temporal flower development might be orchestrated by a diverse set of different metabolites, which are formed by a complex interplay of the different CYP94-enzymes. In order to evaluate this idea and to identify the potential metabolic pathways affected by CYP94 catalysis we used a non-targeted metabolic fingerprinting approach to identify pathways being affected in the flowers of the different *CYP94*-single, double, triple and quadruple mutants. Using this approach we identified the unconjugated form of 12-carboxy-JA-Ile, 12-carboxy-JA, as a novel metabolite that is synthesized in floral tissue exclusively and which has not been detected before (Figs 5 and 7). Notably, besides this compound the only further jasmonate derivative that we could detect in open flowers was 12-carboxy-JA-Ile. The existence of the latter compound in flowers was recently also reported by Widemann and co-workers [14]. The authors demonstrated that 12-carboxy-JA-Ile is highly abundant in flowers and that it is formed from JA-Ile *via* the intermediate formation of 12-hydroxy-JA-Ile upon flower opening. Analysis of T-DNA insertion lines further illustrated that CYP94C1 is a key-player in this process [14]. Our experiments support this hypothesis as we also found that only CYP94C1 deficient plants lack 12-carboxy-JA-Ile. In addition, we found that those plants were also unable to synthesize 12-carboxy-JA. Two possible ways may exist by which this compound can be formed: Either *via* deconjugation of 12-carboxy-JA-Ile by the action of distinct amido hydrolases or *via* direct oxidation of 12-hydroxy-JA by CYP94C1. Although our data on the jasmonate analysis of the different mutants presented above suggest that a direct hydroxylation of JA may occur *in planta* upon wounding in leaves, it is questionable whether the further oxidation of the unconjugated 12-hydroxy-JA is also possible. As mentioned above, we did not detect 12-carboxy-JA in wounded leaves. Moreover, the fact that no 12-hydroxy-JA accumulation could be detected in flowers of CYP94C1 deficient plants, argues against the existence of a “direct” oxidation of 12-hydroxy-JA in flowers. A probably more adequate explanation for the absence of 12-carboxy-JA specifically in *cyp94c1* mutant plants is the nearly complete depletion of the 12-carboxy-JA-Ile pool in those plants. This pool may provide the substrate for distinct amido hydrolases that are capable of cleaving the amino acid from the 12-carboxy-JA-moiety. One potential candidate that might catalyze this reaction in flowers is IAR3. A recent study investigating the recombinant enzyme demonstrated that this amino hydrolase has the capacity to cleave 12-carboxy-JA-Ile, albeit the fact that the specific activity for this substrate was approx. 10-fold lower compared to the activity for the respective 12-hydroxy-derivative [6]. Expression analysis of this enzyme employing the Arabidopsis eFP-Browser indicated that IAR3 is indeed expressed in floral tissues during flower state 12–15 [40].

### Defunctionalization of CYP94B1 and CYP94C1 leads to accumulation of JA-unrelated metabolites that might be characteristic for plant stress response

A further metabolite that was differentially formed by the mutant plants was identified as N-acetyl-amino adipate. This N-acetylated amino dicarboxylic acid was exclusively detected in mutant plants deficient in CYP94B1 suggesting that its catabolic fate is affected by this enzyme. N-Acetyl-amino adipate was originally discovered in the urine of patients suffering from α-amino adipate- and α-keto adipate aciduria [29]. However, its metabolic function in mammals is still elusive. Although this compound has not been observed in plants, its non-acetylated form -α-amino adipiate- is a known metabolite of the lysine catabolic pathway. This pathway is activated upon pathogen infection and leads to the accumulation of α-amino adipate and pipecolic acid [41]. The latter compound is a regulator of systemic acquired resistance [42]. In leaves of unstressed plants the levels of both compounds are extremely low and hardly detectable [42]. The observation that the loss of CYP94B1 activity induces the accumulation N-acetyl-amino adipate may suggest that those plants suffer from severe stress that is probably caused by a disturbed jasmonate homeostasis. This idea is supported by the finding that the triple and quadruple mutants accumulate proline (Fig 5). It is known that different biotic and abiotic stresses can induce proline accumulation in plant tissues [43]. Recently, it has also been demonstrated that proline is highly enriched in floral tissues by approx. a factor of 14 compared to leaves [44]. In addition, proline is suggested to serve as a flowering signal as it influences flowering time and number of flowers [45]. Like this, it might be possible that events further downstream of the CYP94s could have led to the accumulation of new compounds in the flowers metabolism as identified using the non-targeted approach. It is important to note in this context that we detected formation of distinct N-acetylated amino dicarboxylic acids (N-acetyl aminoadipate, and N-acetyl aspartate and glutamate derivatives) only in plant lines with an impaired CYP94B1 and CYP94C1-functionality. The observed chemotypes correlate essentially with the expression profiles of those genes in floral tissues. The chemical properties of N-acetylated amino dicarboxylic acids point to a potential function of these metabolites as chelators. Thus, one may speculate that that these compounds provoke an imbalance in the homeostasis of bivalent cations in the CYP94B1 and CYP94C1-plant lines during flower development.

## Conclusion

In the present study, we found further evidence that the oxidative fate of JA-Ile may be determined by its sequential ω-oxidation which is catalyzed by members of the CYP94-family: CYP94B1, CYP94B2, CYP94B3 and CYP94C1. Although these enzymes exhibit distinct enzymatic activities they can also act semi-redundantly and substitute for each other. Results from the analysis of the quadruple mutant lacking all four CYP94 functionalities (*cyp94b1xcyp94b2xcyp94b3xcyp94c1*) indicate that besides the known and “classical” ω-oxidation of conjugated JA-Ile also a “direct” ω-oxidation of its unconjugated form may occur *in planta*. However, we cannot exclude that this product is formed from 12-hydroxy-JA-Ile by action of specific amido-hydrolases like ILL6 and/or IAR3. In order to tackle this issues experiments are on the way employing mutant plant lines lacking those enzymes (i.e. *cyp94b1xcyp94b2xcyp94b3xcyp94c1xiar3xill6)*.

In addition, our studies revealed that CYP94-functionality influences the temporal development of Arabidopsis flowers. A non-targeted metabolite fingerprinting analysis of floral tissues of plants lacking distinct CYP94-functionalities further indicated that deletion of these enzymes not only affects JA-homeostasis but also additional metabolic pathways. Although the deletion of CYP94B1, for example, only led to small alterations in the amount of different JA-derivatives in wounded leaves, a strong accumulation of metabolites was observed in floral tissues that has not been reported in the context of JA-metabolism and may be characteristic for plant stress responses like systemic acquired resistance. This led us to hypothesize a connection of JA-homeostasis and other metabolic networks (*e*.*g*. of acetylated amino dicarboxylic acids) via a yet unknown link. The identification of this link might provide new insights in the metabolic regulation of plant defense and plant development and will be an exciting topic for further investigations.

## Supporting Information

S1 Fig Confirmation of gene-specific knock-outs of different *CYP94-*mutant lines by semi-quantitative RT-PCR. Analysis was performed by semi-quantitative RT-PCR optimized to 35 PCR cycles. Whereas leaves were used to confirm the knock-out of *CYP94B1*, *CYP94B3* and *CYP94C1* plant lines, respectively, roots were used for *CYP94B2*. For each experiment Col-0 was used as control.
(TIF)

S2 Fig Phenotypical analyses of Col-0 and *CYP94*-mutant lines of *A*. *thaliana*. Plants were grown under long day conditions (16 h light / 8 h dark) at 22°C. **A**) Pictures show one plant rosette (as an average representative of 12 plants) six weeks after sowing. **B**) Analyses of six-week-old Col-0, *cyp94c1*, *cyp94b1xcyp94b2xcyp94b3*, and *cyp94b1xcyp94b2xcyp94b3xcyp94c1* plants in respect to plant height, number of siliques on the main inflorescence, total number of inflorescence, and number of branches on the main inflorescence. Mean values were calculated from ≥ 49 plants ± SD each for two independent experiments.
(TIF)

S3 Fig Flowering time of Col-0 and *CYP94*-mutant lines of *A*. *thaliana*. Plants were grown under long day conditions (16 h light / 8 h dark) at 22°C. Flowering time was estimated by counting the leaves, when inflorescence reached a length of 1 cm. Mean values were calculated from ≥ 18 plants ± SD each of two independent experiments. Letters indicate whether the respective mean values are significantly different as determined by the analysis of variance employing the Tukey post-hoc test.
(TIF)

S4 Fig Flower morphology of Col-0 and *CYP94*-mutant lines of *A*. *thaliana*. Plants were grown under long day conditions (16 h light / 8 h dark) at 22°C. Flowers were collected from six-week-old plants one hour after beginning of the light period. Flowers (stage 13–14) are shown in top-view and side-view as well as with one petal detached for visualization of stamen-length compared to gynoecium length. Data shown are representative for two independent experiments.
(TIF)

S5 Fig Expression analysis of *CYP94*-genes in different tissues of *A*. *thaliana*. Analysis was performed by semi-quantitative RT-PCR optimized to 35 PCR cycles. Tissues were harvested in mid-flowering state, seven weeks after sowing (long day). The analysis was performed twice with material from independent Col-0 plants.
(TIF)

S6 Fig Expression profile of promoter:GUS constructs for CYP94B1, CYP94B2, CYP94B3 and CYP94C1 in reproductive organs of *A*. *thaliana*. Transformed plants were grown on soil under long-day (16 h light / 8 h dark) conditions for four weeks. All plant lines were stained with 2 mM X-Gluc. Staining was performed with two independent plant lines per construct.
(TIF)

S1 Table Primers used for genotyping of the different plant lines. (DOCX)

S2 Table Results from the Tukey post-hoc test of the one-way analysis of variance (ANOVA). The analysis was performed by using the Origin Pro 8.5 software and is presented as an Excel-file. Mean-values that were significantly different (*p*<0.05) are shaded yellow.
(XLSX)

S3 Table Data matrix of 164 high quality features (FDR < 10^−4^) obtained by metabolite fingerprinting of flowers of *A*. *thaliana* Col-0 and *cyp94b1*, *cyp94b2*, *cyp94b3*, *cyp94c1* single, double, triple and quadruple mutant lines (see Fig 5A). (XLSX)
