# Supplementary figures and images for: Functional Characterization of CYP94-Genes and Identification of a Novel Jasmonate Catabolite in Flowers
Source: PLoS One. 2016 Jul 26;11(7):e0159875. doi: 10.1371/journal.pone.0159875 (PMC4961372; doi:10.1371/journal.pone.0159875)

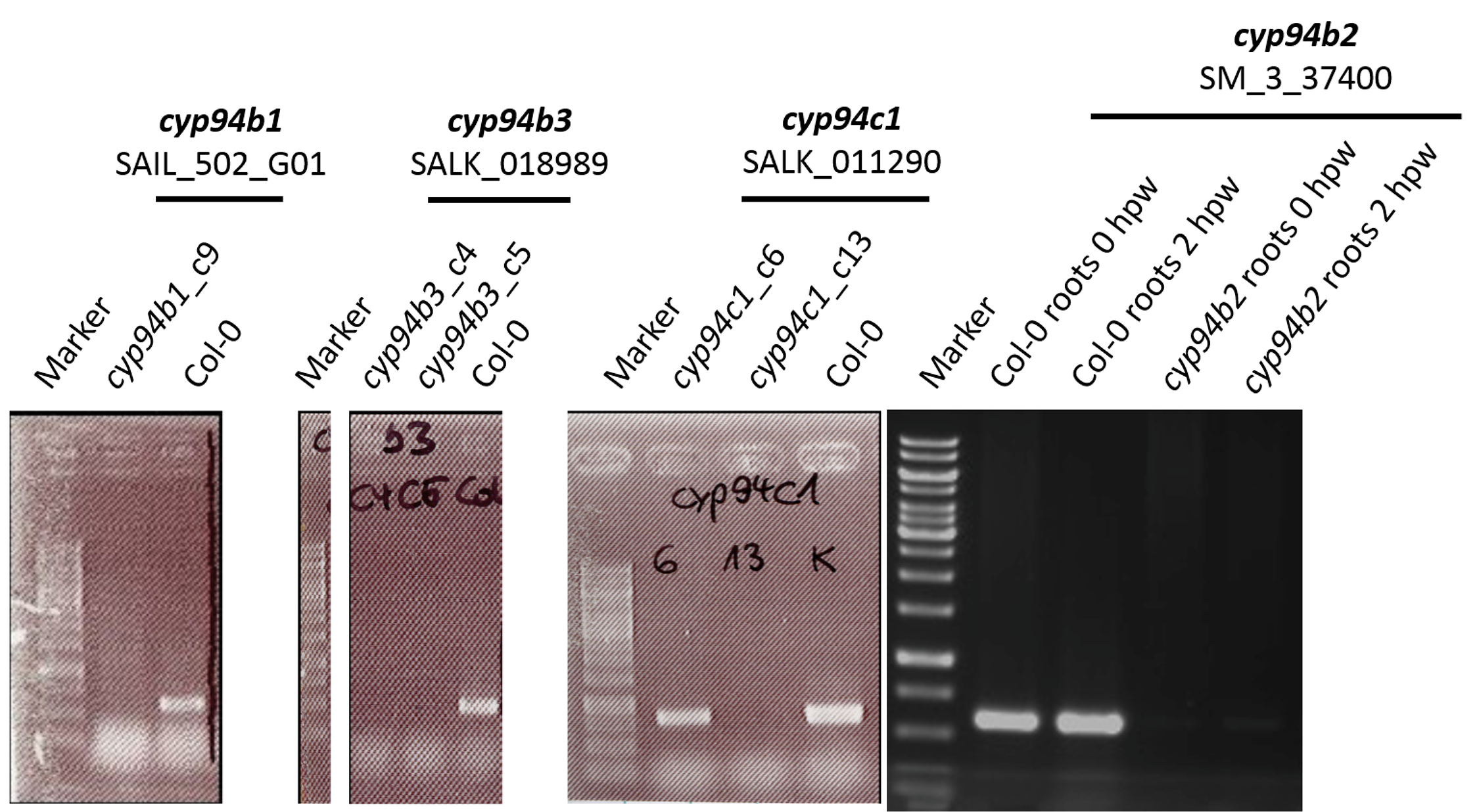

Supplement: S1 Fig — Analysis was performed by semi-quantitative RT-PCR optimized to 35 PCR cycles. Whereas leaves were used to confirm the knock-out of CYP94B1, CYP94B3 and CYP94C1 plant lines, respectively, roots were used for CYP94B2. For each experiment Col-0 was used as control. (TIF) [file pone.0159875.s001.tif]

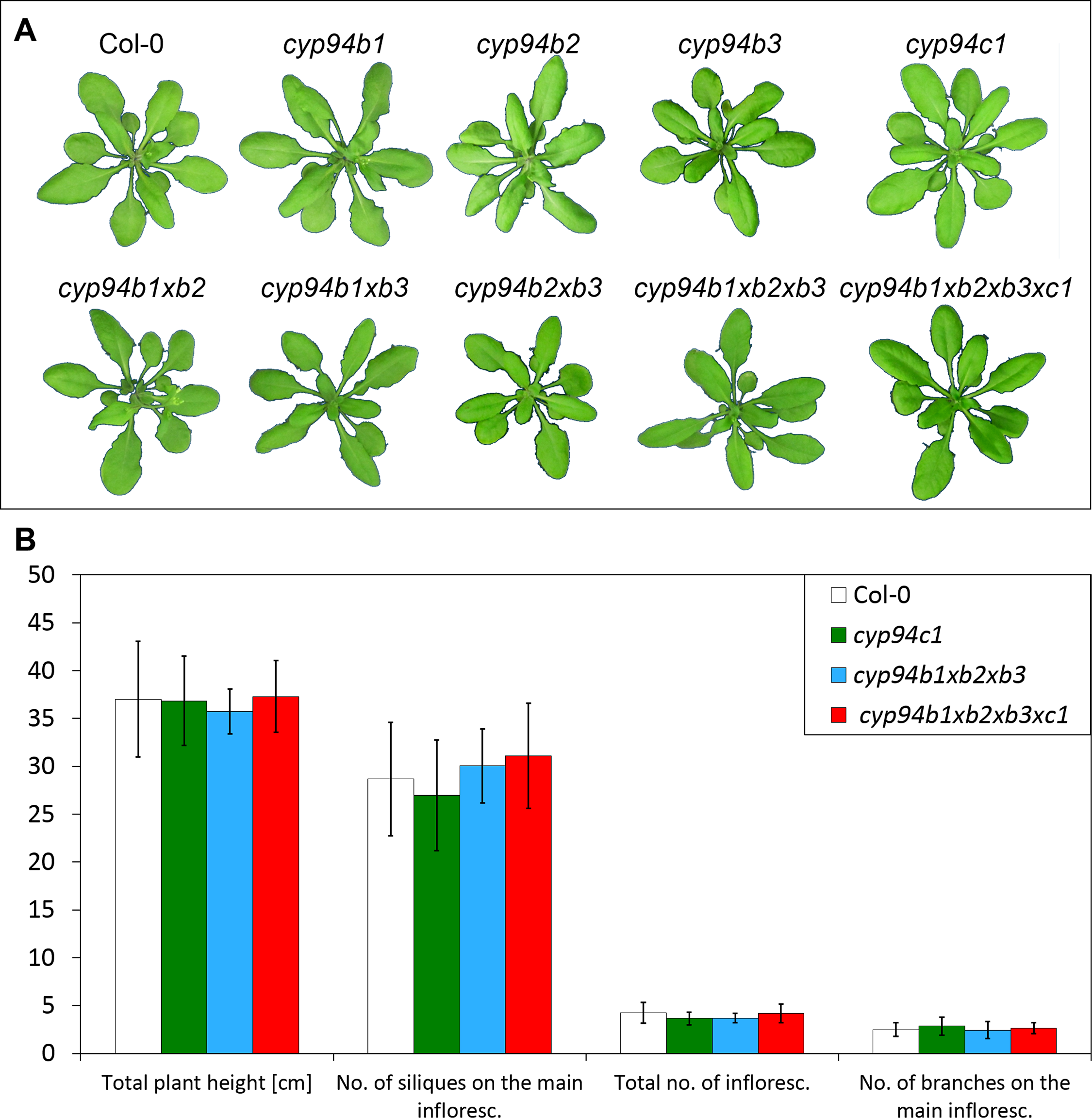

Supplement: S2 Fig — Plants were grown under long day conditions (16 h light / 8 h dark) at 22°C. A) Pictures show one plant rosette (as an average representative of 12 plants) six weeks after sowing. B) Analyses of six-week-old Col-0, cyp94c1, cyp94b1xcyp94b2xcyp94b3, and cyp94b1xcyp94b2xcyp94b3xcyp94c1 plants in respect to plant height, number of siliques on the main inflorescence, total number of inflorescence, and number of branches on the main inflorescence. Mean values were calculated from ≥ 49 plants ± SD each for two independent experiments. (TIF) [file pone.0159875.s002.tif]

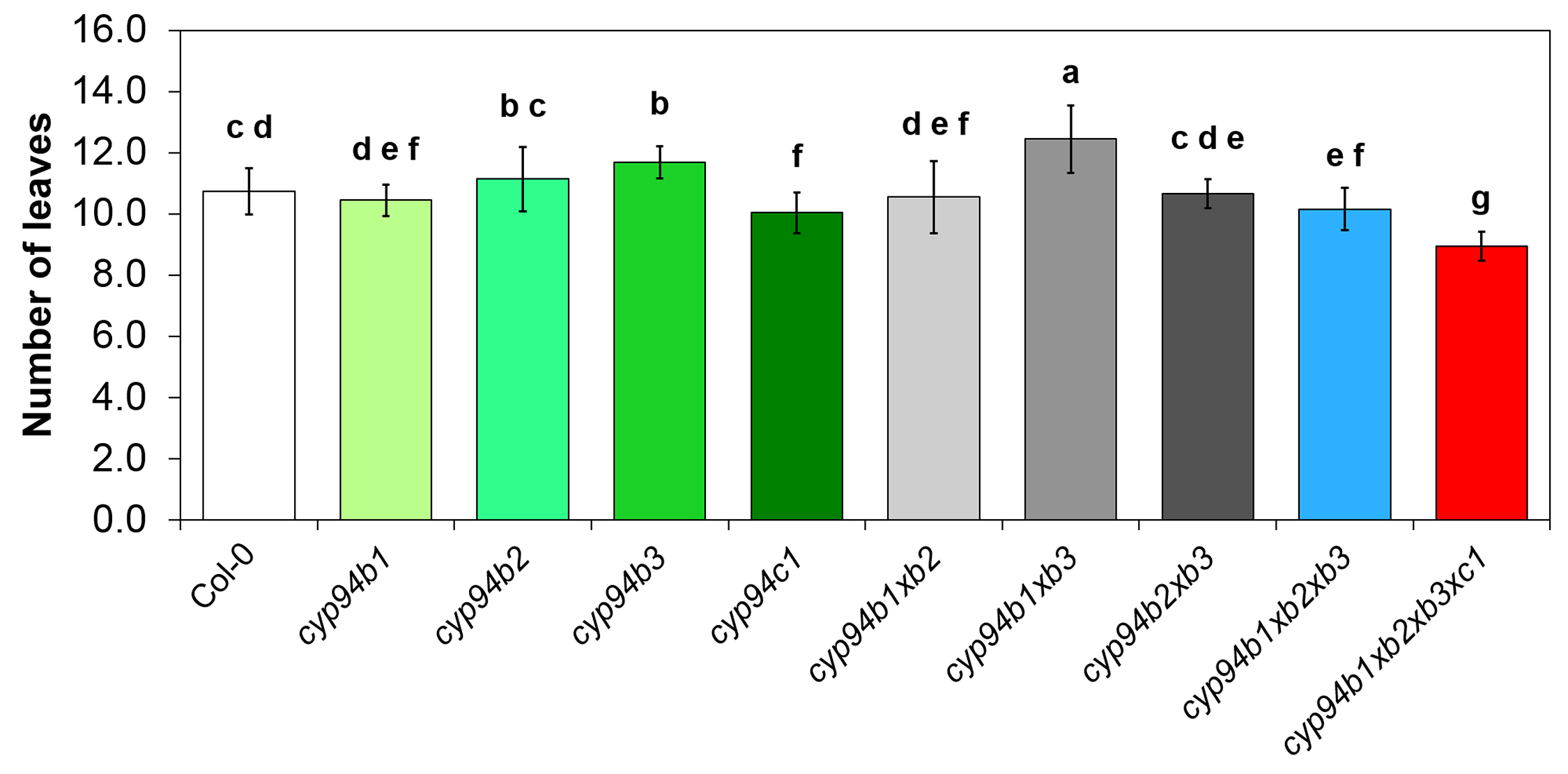

Supplement: S3 Fig — Plants were grown under long day conditions (16 h light / 8 h dark) at 22°C. Flowering time was estimated by counting the leaves, when inflorescence reached a length of 1 cm. Mean values were calculated from ≥ 18 plants ± SD each of two independent experiments. Letters indicate whether the respective mean values are significantly different as determined by the analysis of variance employing the Tukey post-hoc test. (TIF) [file pone.0159875.s003.tif]

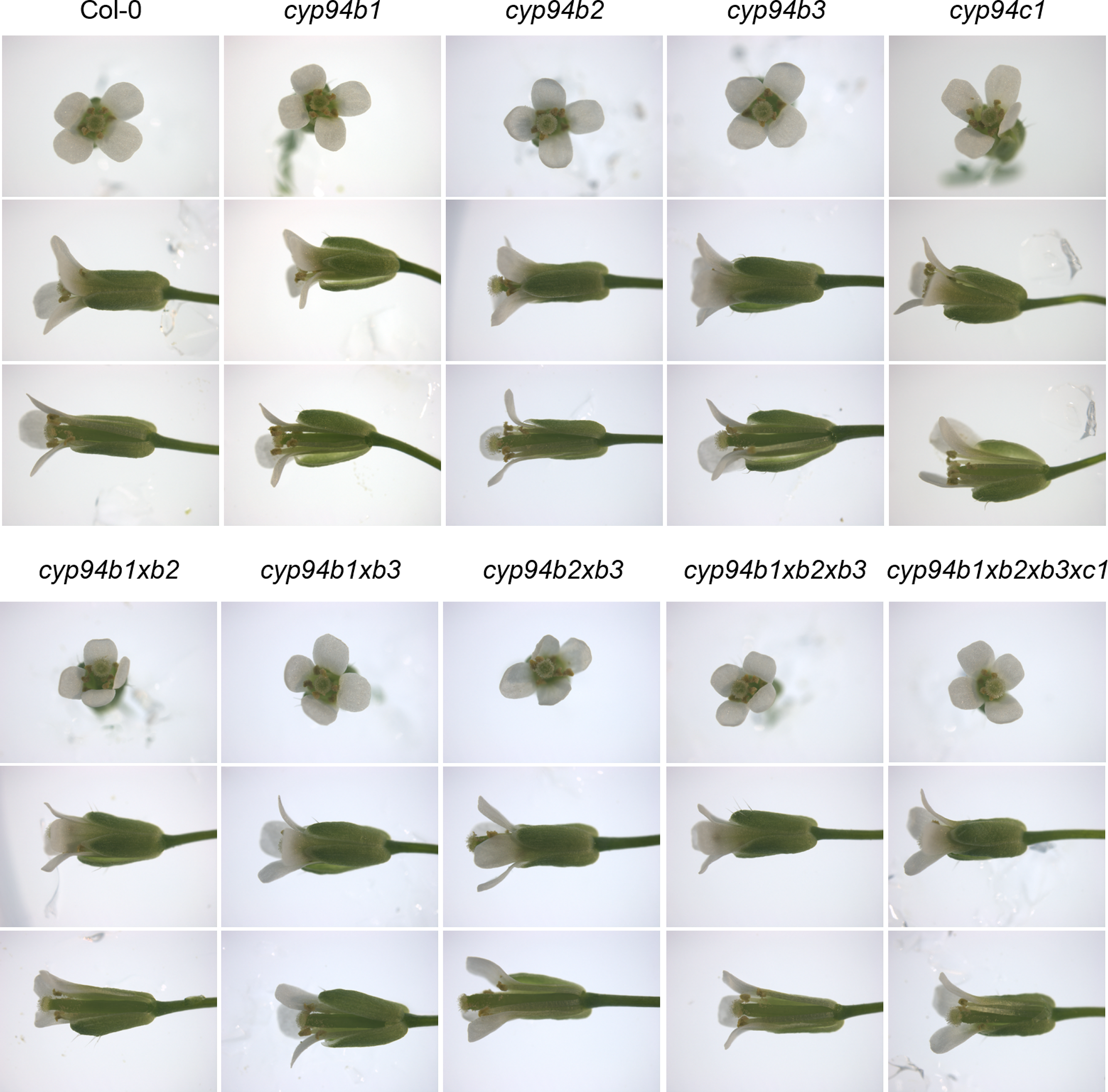

Supplement: S4 Fig — Plants were grown under long day conditions (16 h light / 8 h dark) at 22°C. Flowers were collected from six-week-old plants one hour after beginning of the light period. Flowers (stage 13–14) are shown in top-view and side-view as well as with one petal detached for visualization of stamen-length compared to gynoecium length. Data shown are representative for two independent experiments. (TIF) [file pone.0159875.s004.tif]

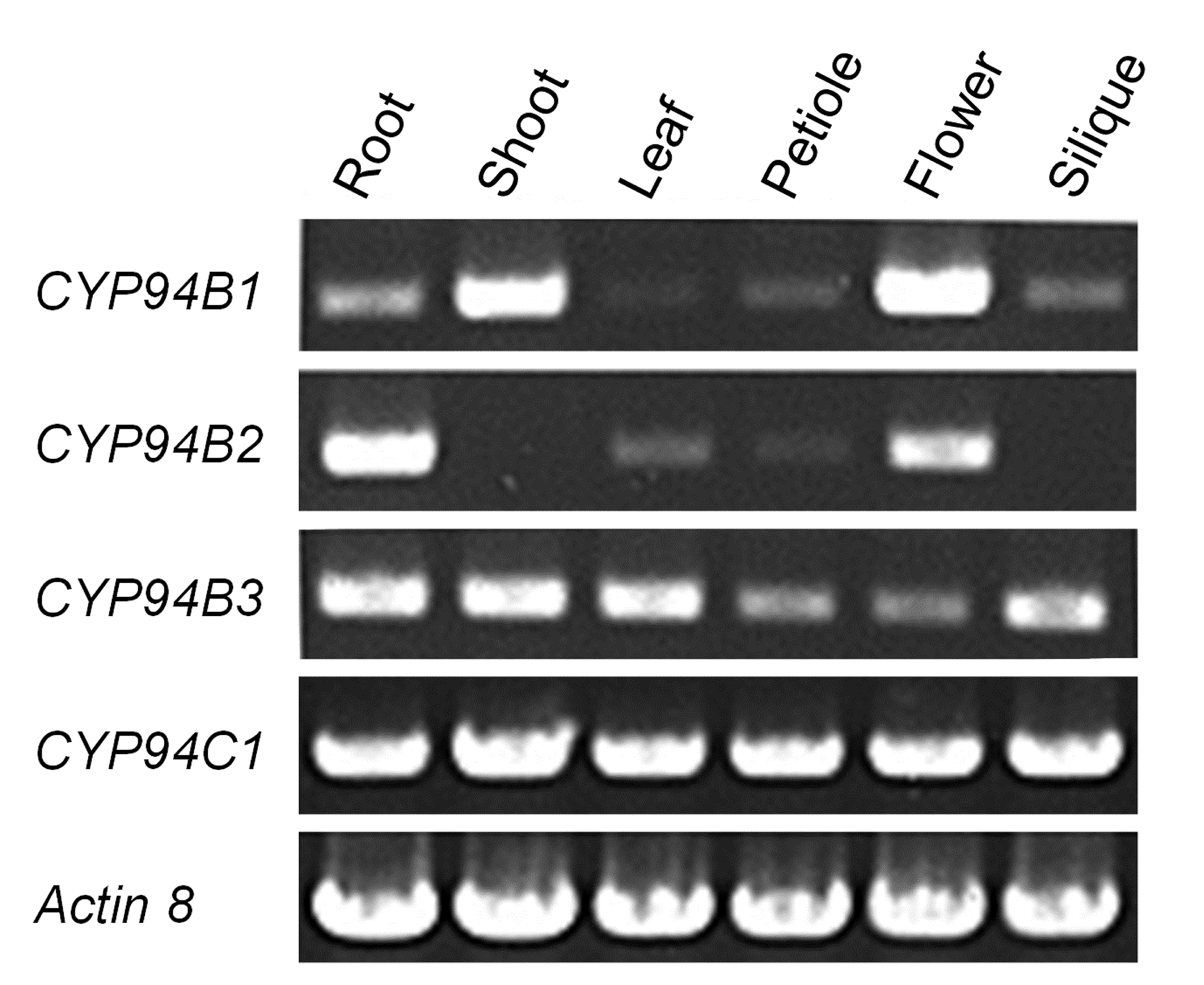

Supplement: S5 Fig — Analysis was performed by semi-quantitative RT-PCR optimized to 35 PCR cycles. Tissues were harvested in mid-flowering state, seven weeks after sowing (long day). The analysis was performed twice with material from independent Col-0 plants. (TIF) [file pone.0159875.s005.tif]

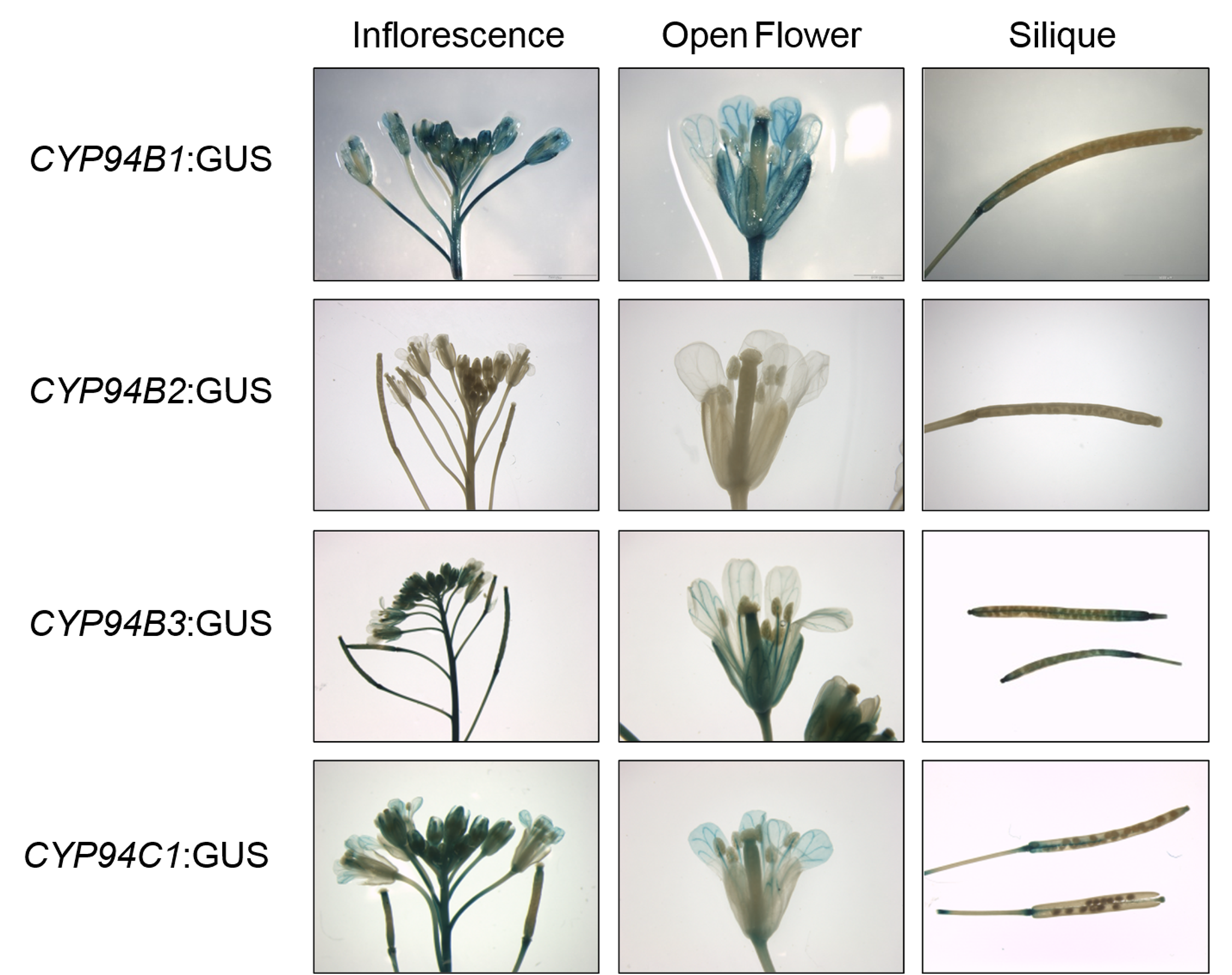

Supplement: S6 Fig — Transformed plants were grown on soil under long-day (16 h light / 8 h dark) conditions for four weeks. All plant lines were stained with 2 mM X-Gluc. Staining was performed with two independent plant lines per construct. (TIF) [file pone.0159875.s006.tif]
