# Supplementary material for: Functional Characterization of CYP94-Genes and Identification of a Novel Jasmonate Catabolite in Flowers
Source: PLoS One. 2016 Jul 26;11(7):e0159875. doi: 10.1371/journal.pone.0159875 (PMC4961372; doi:10.1371/journal.pone.0159875)
Supplement: S1 Table — (DOCX) [file pone.0159875.s007.docx]

S1 Table: Primers used for genotyping of the different plant lines

| Primer name | Nucleotide sequence (5‘->3‘) |
| --- | --- |
| SAIL_502_G01_LP (cyp94b1) | CCTAAAGGCAAACCTTTCACC |
| SAIL_502_G01_RP (cyp94b1) | CATGTGAGCGGTTAGAAGAGG |
| SM_3_37400_LP (cyp94b2) | TCAAGGGGAAAGAGAAGGATC |
| SM_3_37400_RP (cyp94b2) | GCAAACCTTTTACGGAGATCC |
| SALK_018989_LP (cyp94b3) | GATCTTCACCTTAAGCCCACC |
| SALK_018989_RP (cyp94b3) | CGTCTAGTCCCGGTTTTATCC |
| SALK_011290_LP (cyp94c1) | AAACTCAGATTCTTCAATCCGC |
| SALK_011290_RP (cyp94c1) | TGATAAACCGGTTAGCTGGTG |
